# Integrated transcriptome analysis for the hepatic and jejunal mucosa tissues of broiler chickens raised under heat stress conditions

**DOI:** 10.1186/s40104-022-00734-y

**Published:** 2022-07-18

**Authors:** Deok Yun Kim, Byeonghwi Lim, Jun-Mo Kim, Dong Yong Kil

**Affiliations:** Department of Animal Science and Technology, Chung-Ang University, Anseong-si, Gyeonggi-do 17546 Republic of Korea

**Keywords:** Broiler chicken, Heat stress, Jejunal mucosa, Liver, Transcriptome

## Abstract

**Background:**

Heat stress (HS) is one of the most important threats for the current poultry industry. Therefore, many efforts have been made to ameliorate the adverse effect of HS on poultry production; however, physiological and molecular mechanisms pertaining to HS are still limited in poultry. Therefore, the objective of the current study was to investigate functional alterations based on individual and integrated transcriptomes in the liver and jejunal mucosa tissues of broiler chickens exposed to HS conditions.

**Results:**

Broiler chickens exposed to HS showed decreased growth performance and increased corticosterone concentrations in the feather. In the transcriptome analysis, the number of differentially expressed genes (DEGs) were identified in the liver and jejunal mucosa by HS conditions. In the liver, genes related to amino acid oxidation, tryptophan metabolism, lipid metabolism, oxidative phosphorylation, and immune responses were altered by HS, which support the reason why heat-stressed poultry had decreased productive performance. In the jejunal mucosa, genes related to defense systems, glutathione metabolism, detoxification of xenobiotics, and immune responses were differently expressed by HS conditions. The integrated transcriptome analysis with DEGs found in the liver and jejunal mucosa showed a considerable connectivity between the core nodes in the constructed networks, which includes glutathione metabolism, xenobiotic metabolism, carbon metabolism, and several amino acid metabolisms.

**Conclusions:**

The core network analysis may indicate that increased requirement of energy and amino acids in the jejunal mucosa of broiler chickens exposed to HS conditions is likely compromised by increased oxidation and synthesis of amino acids in the liver. Therefore, our results may provide comprehensive insights for molecular and metabolic alterations of broiler chickens raised under HS conditions, which can aid in the development of the novel strategies to ameliorate the negative effect of HS on poultry productivity and health.

**Supplementary Information:**

The online version contains supplementary material available at 10.1186/s40104-022-00734-y.

## Background

Environmental temperature in the world constantly increases due to the global warming. This global warming is one of the most considerable threats for the current livestock industry because heat stress (HS) largely decreases animal productivity and health. Poultry is known to be the most sensitive to HS among livestock animals because poultry is covered with feathers and has no sweat glands, which impairs heat dissipation in the body [1]. In addition, the recent intensive breeding scheme of poultry realizes very high performance of poultry; however, it concomitantly causes an increase in body heat production, which can worsen the temperature regulation of poultry [2]. Therefore, many poultry researchers currently focus on the development of novel strategies to ameliorate HS of poultry. Among various strategies, dietary modifications are the most widely studied because they are easy to be adopted and practical in the commercial poultry production [3–6]. However, few successful strategies regarding dietary modifications have been identified. One of possible reasons may be the limited information for the effects of HS on physiological and metabiological alterations in poultry.

The RNA sequencing (RNA-Seq) technology is widely used for analyzing overall transcriptomic changes in the pivotal body tissues as affected by various internal and external factors. Thus, the transcriptomic analysis based on RNA-Seq can contribute to an improvement in the current knowledge of molecular and functional mechanisms underlying physiological changes in poultry exposed to HS conditions. Previous studies using RNA-Seq have revealed that HS resulted in increased expression of genes related to various nutrient metabolism and heat shock proteins in the liver of poultry [7, 8]. Moreover, Bertocchi et al. [9] conducted the transcriptomic analysis of intestinal tissues of broiler chickens raised under HS conditions and reported increased production of reactive oxygen species (ROS) but decreased oxidative phosphorylation, amino acid metabolism, and immune responses. The liver plays a role in regulating nutrient metabolism, hormone production, detoxification, immune system, and circulatory protein levels [10]. Likewise, the small intestine is the central organ for nutrient digestion and absorption to provide the liver with dietary nutrients [11]. In addition, intestinal tissues play a role in body defense systems to protect from the entrance of harmful materials such as toxins and pathogens from the lumen to the body [12], which is also highly associated with liver health and function. Therefore, physiological connection and crosstalk between the liver and small intestine are expected to be substantial. Therefore, the integrated analysis of the functions of these tissues as affected by HS conditions is essential to reveal physiological and metabolical alterations in heat-stressed poultry. However, to our knowledge, there have been no studies pertaining to integrated transcriptomic analysis between these pivotal tissues of poultry, particularly raised under HS conditions.

The objective of the current study, therefore, was to investigate changes in tissue-specific transcriptomes in the liver and jejunal mucosa of broiler chickens exposed to HS conditions, and further to search for genes and their functional communications in the core network between the liver and jejunal mucosa based on the integrated transcriptomic analysis.

## Materials and methods

### Birds and experimental design

The research protocol for the present experiment was approved by the Institutional Animal Care and the Use Committee (IACUC) at Chung-Ang University (approval number: 2019–00086). A total of five hundred 1-day-old Ross 308 male broiler chicks was obtained and raised according to a general guideline of Ross 308 broiler management until 20 days of age. All chicks were fed a commercial diet adequate in energy and nutrients [13] before the start of the experiment. On 21 days of age, broiler chickens (660 ± 2.39 g) were weighed and two hundred broiler chickens with narrow body weight (BW) were finally selected. The selected chickens were allocated to 20 battery cages, which were randomly assigned to 2 different temperature groups of thermoneutral zone (TN) and HS conditions with 10 replicates per group. For TN conditions, birds were raised at 22 °C and 60% relative humidity (RH) during 21–35 days of age, according to the Ross 308 broiler management guide [13]. For HS conditions, cyclic HS conditions were adopted. The birds raised under HS conditions were kept at 32 °C and 70% RH during 21–28 d and 30 °C and 70% RH during 29–35 days of age from 10:00 to 18:00 h. For the remaining time, the room temperature was reduced to 26 °C with 60% RH during 21–28 d and 24 °C and 60% RH during 29–35 days of age.

A common and commercial-type diet containing an adequate amount of energy and nutrients (apparent metabolizable energy = 3200 kcal; crude protein = 19.5%) for broiler chickens [13] were prepared and fed to all birds throughout the experiment. The feed and water were provided ad libitum throughout the experiment. The BW, BW gain (BWG), and feed intake (FI) were recorded at the end of the experiment. The feed efficiency (FE) was calculated as BWG divided by FI after the correction of mortality [14].

### Feather corticosterone extraction and assay

At the conclusion of the experiment (i.e., 35 days of age), to validate the effects of HS conditions to stress responses in broiler chickens, one bird with the average BW per replicate was selected (i.e., 10 birds per treatment) to analyze feather corticosterone (CORT) concentrations as a stress indicator [15]. Feather samples were collected from the primary flight feather and were stored at − 80 °C before analysis. A methanol-based extraction technique was used to extract CORT from the feathers [16, 17]. The calamus was removed at the time of sample collection. Except for the calamus, the feather vane was cut with scissors into pieces of < 5 mm^2^. The sheared feathers with 10-mL methanol (HPLC grade, Honeywell) were placed in a sonicating water bath at room temperature for 30 min, followed by incubation in a shaking water bath at 50 °C for overnight. The methanol was subsequently separated from feather materials by 0.45 μm syringe filter (HyunDai Micro, Anseong, Korea). The feather residues were treated again with a syringe filter by adding 2 mL of methanol twice; the washes were injected into the original methanol extract. The methanol extract was set in a 50 °C water bath and evaporated in a fume hood. After the completion of evaporation of the sample, the remaining extract were mixed in a 1 mL of the phosphate buffer system (0.01 mol/L and pH 7.4) for reconstituting the amount. The filtration step was found to be enough to remove the feather residue, but if the feather residue remains severely, it is separated using a centrifuge. Reconstituted samples were stored at − 80 °C until CORT analysis. Feather CORT concentrations were analyzed using the Corticosterone Competitive ELISA kit (Thermo Fisher scientific, USA).

### Sample preparation for RNA-Seq

At the end of experiment (i.e., 35 days of age), additional one bird with similar BW to average BW per replicate was chosen (i.e., 10 birds per treatment) after 8-h fasting. However, 2 birds with the least or greatest BW were discarded before the final analysis to obtain more homogenous bird groups. Finally, a total of 16 birds (TN: 8 birds and HS: 8 birds) were selected and euthanized by CO_2_ asphyxiation. Liver and jejunal mucosa samples were immediately collected, snap-frozen using a liquid nitrogen, and stored at − 80 °C for the later total RNA isolation.

### RNA isolation, library construction, and RNA-Seq

Total RNA was isolated from the liver and jejunal mucosa tissues using TRIzol reagent (Invitrogen, Life Technologies, Carlsbad, CA, USA) according to the manufacturer’s recommendations. Quality check of the total RNA was performed with RNA-integrity number (RIN; Additional file 1: Table S1). Total RNA was synthesized as cDNA for library constructions. The constructed libraries were analyzed using an Illumina HiSeq X Ten instrument (Illumina, Inc., San Diego, CA, USA) and paired-end (2 × 150 base pair) sequencing was conducted. Detailed procedures for RNA sequencing were reported previously [18].

### Data processing and differentially expressed gene (DEG) analyses

The quality of raw read data for each sample were checked using the FastQC software v0.11.7. The reads were trimmed with adaptors using the Trimmomatic v0.38 based on the quality results. Thereafter, the trimmed reads were mapped to the reference genome (GRCg6a, GCA_000002315.5) of the Ensembl genome browser [19] using HISAT2 v2.1.0. Raw counts in each library were computed based on the exons in *Gallus gallus* GTF v100 (Ensembl) using the featureCounts of Subread package v1.6.3. Whole DEG analyses for the obtained raw counts were performed using the edgeR v3.26.5 and the raw counts was normalized using the trimmed mean of M-value (TMM) method. The DEGs were identified for the liver and jejunal mucosa of broiler chickens raised under HS vs. TN conditions. The false discovery rate (FDR) of < 0.05 and an absolute log_2_ fold-change of ≥1 were adopted. Multidimensional scaling (MDS) was carried out to demonstrate the closeness among samples per tissue. More detailed methods are previously described by Lim et al. [18].

### Functional enrichment analyses

The DEGs were annotated to the gene ontology (GO) terms and Kyoto Encyclopedia of Genes and Genomes (KEGG) pathways using Database for Annotation, Visualization, and Integrated Discovery (DAVID) v6.8. The GO enrichment analyses were performed with biological process (BP), cellular component (CC), and molecular function (MF) with the following cut-offs: *P*-value < 0.1 and counts ≥2. Enriched GO terms were bound with similar terms and visualized with treemaps using the REVIGO. The most significant GO terms in the bound group were shown as a representative. The KEGG enrichment analyses were also conducted using the same cut-off criteria and were represented by the –log_10_ *P*-value and fold enrichment. All enrichment analyses were annotated in a *Gallus gallus* species.

### Gene set-enrichment analyses (GSEA)

The Gene Set-Enrichment Analyses (GSEA) for DEGs was performed using the gene-ranking method based on gene sets in the KEGG database using the GSEA v4.0.2. All analyses were conducted using the normalized TMM counts of the liver and jejunal mucosa tissues. The GSEA results were visualized as bubble plots with significant pathways (*P* < 0.05) and normalized enrichment score.

### Integrative network analyses

The correlation matrix was constructed to integrated functional analysis between DEGs of the liver and jejunal mucosa tissues. Pearson correlation coefficient was used as the basis for a hierarchical clustering with the complete linkage method. Highly positive correlated DEGs’ group between liver and jejunal mucosa tissues included many genes related to energy metabolisms, and these DEGs were annotated to identify their functions using the KEGG database in DAVID. The network based on the annotated KEGG pathway was constructed by Cytoscape v.3.7.1. Afterwards, the modulations of responsible gene products (i.e., proteins) in the two main KEGG pathways that were relevant to each other were confirmed using the clusterProfiler package in the R. The genes indicating the maximum changes among the genes related to each protein were utilized as the representative values.

### Quantitative real-time PCR validation

To verify the reliability of the expression profiles of RNA-seq data, we randomly evaluated expression of twelve each for DEGs by quantitative real-time PCR (qPCR) followed the method of Shin et al. [5]. In short, total RNA was extracted from the liver and jejunal mucosa using TRIzol reagent (Invitrogen, Carlsbad, CA) according to the manufacturer’s instructions. Gene expression was examined for acetyl-CoA carboxylase alpha (*ACACA*), cytochrome P450 family 1 subfamily C polypeptide 1 (*CYP1C1*), fatty acid synthase (*FASN*), heat shock protein family A (Hsp70) member 2 (*HSPA2*), and stearoyl-CoA desaturase (*SCD*) in the hepatic tissues. In the jejunal mucosa, fibroblast growth factor 1 (*FGF1*), glutathione peroxidase 1 (*GPX1*), glutathione S-transferase alpha 3 (*GSTA3*), heat shock protein family A (Hsp70) member 2 (*HSPA2*), and interleukin 1 beta (*IL1B*) were adopted to examine gene expression. Gene-specific primers for target genes were designed using NCBI/Primer-BLAST. The specificity of the primers was confirmed by PCR amplification as demonstrated by Aznar and Alarcón [20]. The primer sequences and amplification temperatures are listed in additional file 1: Table S2. The relative quantification of gene-specific expression was calculated using the 2^-^^ΔΔCt^ method after normalization to glyceraldehyde-3-phosphate dehydrogenase (*GAPDH*) [21].

### Statistical analysis for growth performance and feather corticosterone

All data were analyzed by ANOVA as a completely randomized design using the PROC MIXED procedure (SAS Institute Inc., Cary, NC). The replicated cages were considered as an experimental unit (*n* = 10) for growth performance, whereas individual bird was considered as an experimental unit for feather CORT concentrations. Outlier data were checked using the PROC UNIVARIATE procedure of SAS; however, no outliers were identified. The LSMEANS procedure was used to calculate treatment means. Significance for statistical tests was set at *P* < 0.05.

## Results

### Growth performance and feather corticosterone

**Fig. 1:**
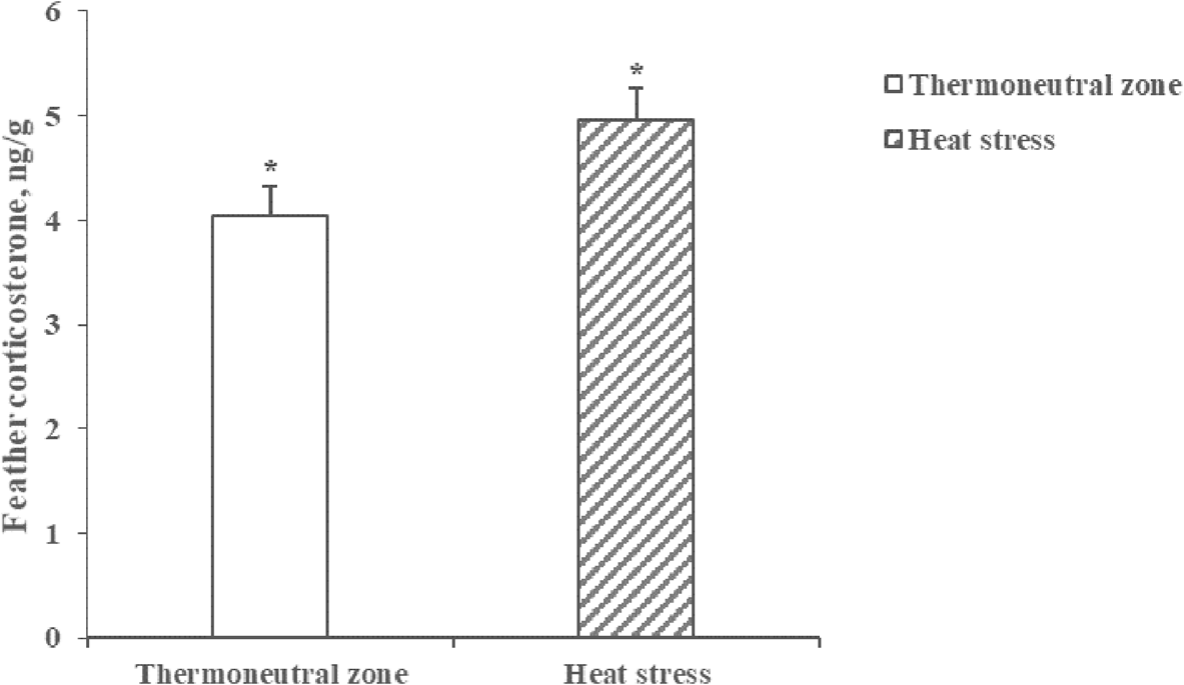
Feather corticosterone concentrations of broiler chickens raised under thermoneutral zone (TN) or heat stress (HS) conditions. Values are means ± SEM (*n* = 10), * indicates *P* < 0.05

During the overall experimental period from 21 to 35 days of age, birds raised under HS conditions had less (*P* < 0.05) growth performance including BWG, FI, and FE compared with birds raised under TN conditions (Table 1). The average feather CORT concentrations were 4.03 ± 0.20 and 4.96 ± 0.29 ng/g for birds raised under TN conditions and HS conditions, respectively. A significant increase (*P* < 0.05) in feather CORT concentrations by 18.8% was observed for birds raised under HS conditions than for those raised under TN conditions (Fig. 1).

**Table 1:** Growth performance of broiler chickens raised under thermoneutral zone (TN) or heat stress (HS) conditions from 21 to 35 days of age

Items^a^	Condition^b^		SEM^c^	*P*-value
	TN	HS		
Final BW, g	2182	1862	37.4	< 0.01
BWG, g	1521	1201	37.1	< 0.01
FI, g	2391	2183	52.5	< 0.05
FE, g/kg	639	550	0.015	< 0.01

Data are least squares means of 10 observations per treatment (*n* = 10)

^a^*BW*, body weight, *BWG*, BW gain; *FI*, feed intake; *FE*, feed efficiency (BWG:FI)

^b^*TN*, thermoneutral; *HS*, heat stress

^c^*SEM*, standard error of the mean

### Data processing and transcriptomes under heat-stressed condition

A total of 661 million paired-end reads were generated from 32 samples (i.e., 2 tissues of 16 chickens). The average of the number of generated reads per sample was 20.7 million and the average trimmed read was 17.3 million (16.2% were trimmed out; Additional file 1: Table S3). Overall mapping rates were ranged from 92.79% to 96.05% and the average unique mapping rate was 84.60%.

**Fig. 2:**
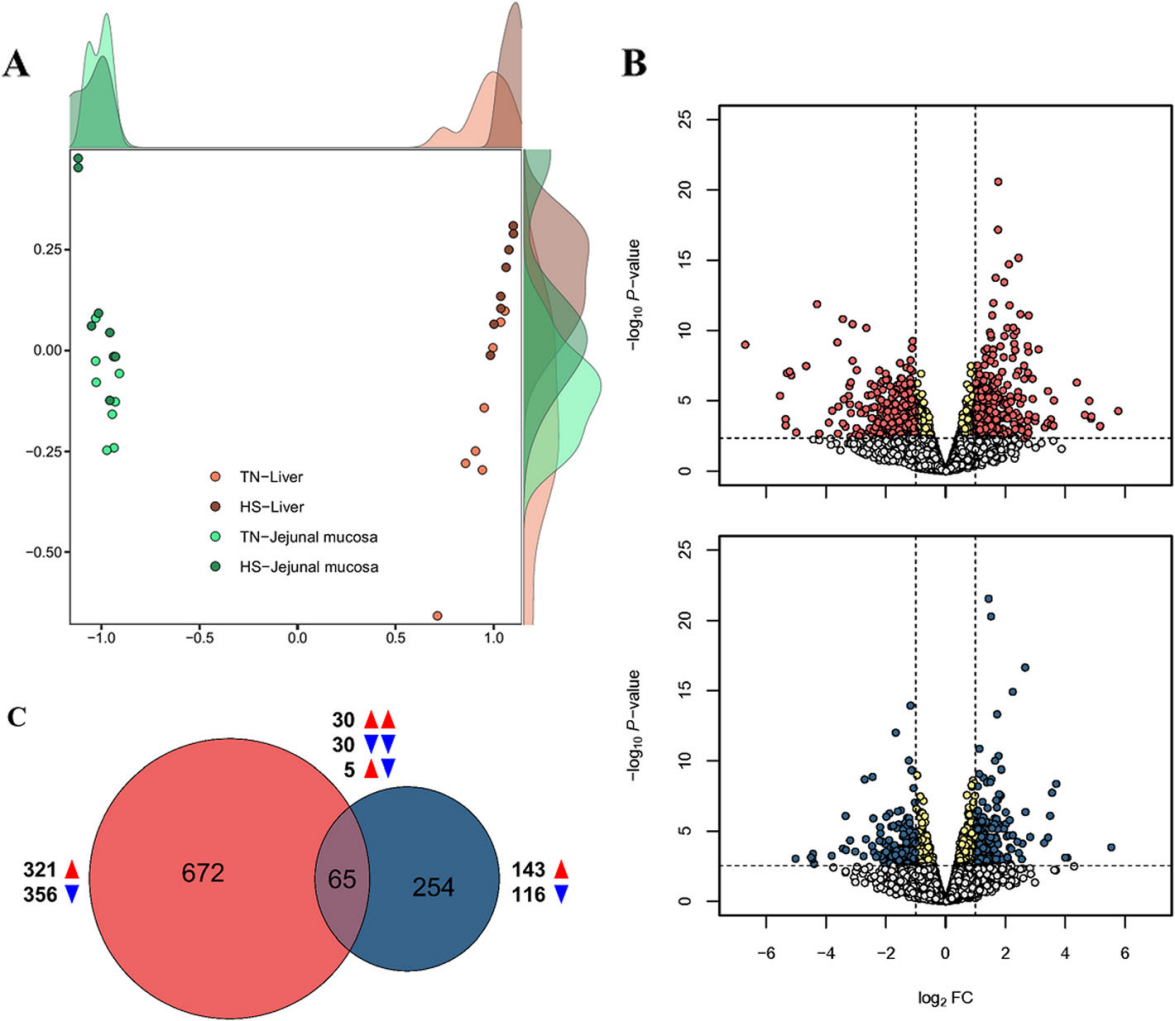
Overall transcriptomes in liver and jejunal mucosa. **A** Multidimensional scaling (MDS) reveals separate clusters between the two tissues based on the transcriptomes under thermoneutral zone (TN) and heat stress (HS). **B** Volcano plots indicating significant differently expressed genes (DEGs) in the liver (red) and jejunal mucosa (blue). The *x* and *y* axes of the volcano plots show the log_2_ fold changes and -log_10_ *P*-values, respectively. **C** Venn diagram is visualized based on overlapping number of DEGs between liver (red) and jejunal mucosa (blue) tissues

The NGS read were generated from 2 tissues (liver and jejunal mucosa) at 35 d including HS conditions (*n* = 8) or TN conditions (*n* = 8). Clear clustering was shown for each tissue in the MDS analysis (Fig. 2A). The analysis of DEGs was performed by comparing gene expression levels in each tissue under HS conditions, based on those in each tissue under TN conditions, which were visualized by volcano plots (Fig. 2B). The number of DEGs in the liver and jejunal mucosa was identified with 737 and 319, respectively. A Venn diagram showed overlapping DEGs between the tissues, consisted of 30 up-regulated, 30 down-regulated genes, and 5 opposite regulated genes (up in the liver and down in jejunal mucosa) among 65 genes between 2 tissues (Fig. 2C).

### Functional annotations in each tissue

**Fig. 3:**
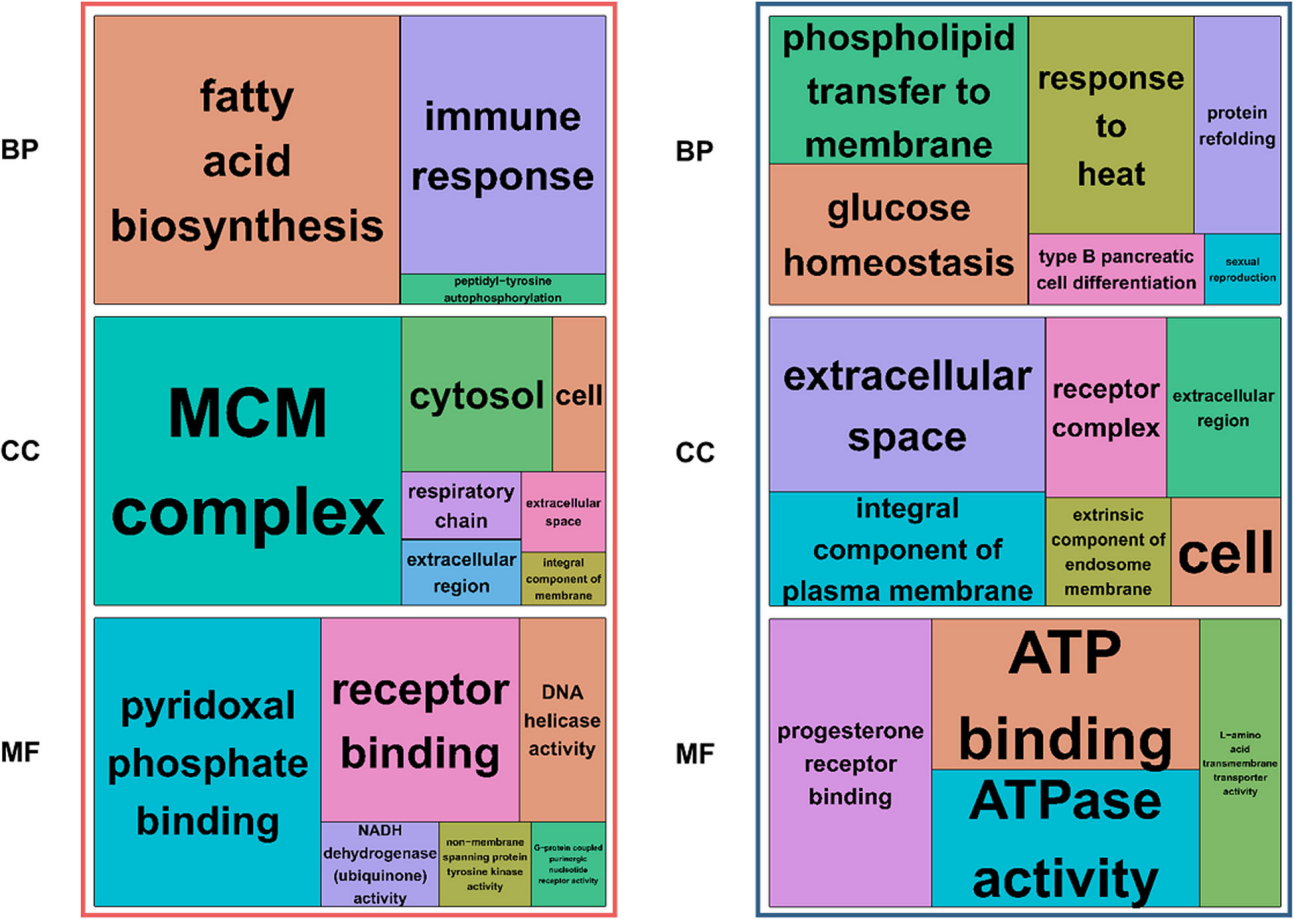
Functional enrichment in liver and jejunal mucosa. The gene ontology treemaps are created based on the *P*-values related to the biological process (BP), cellular component (CC), and molecular function (MF) terms in the liver (red) and jejunal mucosa (blue) tissues

**Fig. 4:**
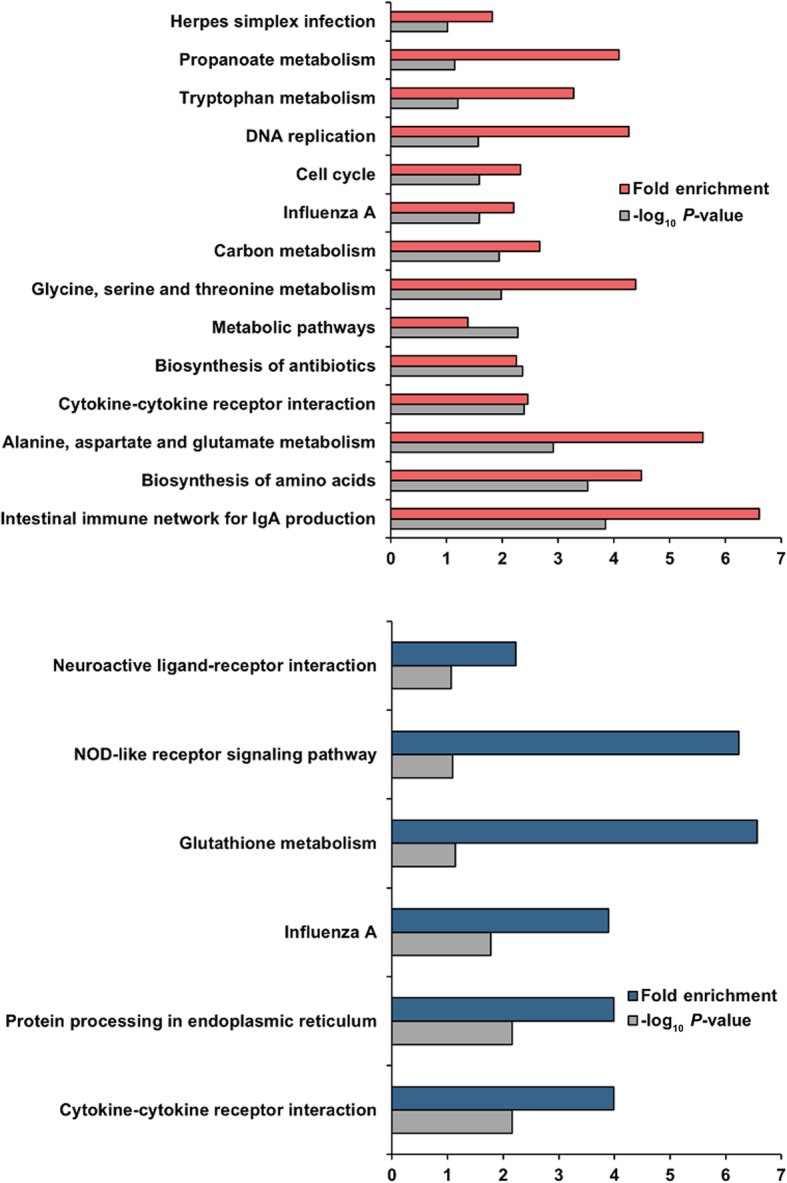
Functional enrichment in liver and jejunal mucosa. The Kyoto Encyclopedia of Genes and Genomes (KEGG)-enriched pathways are generated for the liver (red) and jejunal mucosa (blue) tissues with -log_10_ *P*-values > 1.0

Enrichment analyses were performed based on the GO (Fig. 3) and KEGG (Fig. 4) databases in the liver and jejunal mucosa. The BPs of GO for the liver were significantly enriched to ‘fatty acid biosynthesis’, ‘immune response’, and ‘peptidyl-tyrosine autophosphorylation’. The CCs of GO for the liver were enriched to ‘MCM complex’, ‘cytosol’, ‘cell’, ‘respiratory chain’, ‘extracellular region’, ‘extracellular space’, and ‘integral component of membrane’. The MFs of GO for liver were enriched to ‘pyridoxal phosphate binding’, ‘receptor binding’, ‘DNA helicase activity’, ‘NADH dehydrogenase (ubiquinone) activity’, ‘non-membrane spanning protein tyrosine kinase activity’, and ‘G-protein coupled purinergic nucleotide receptor activity’. Otherwise, the BPs of GO for the jejunal mucosa was enriched to ‘phospholipid transfer to membrane’, ‘glucose homeostasis’, ‘response to heat’, ‘protein refolding’, ‘type B pancreatic cell differentiation’, and ‘sexual reproduction’. The CCs of GO for the jejunal mucosa was enriched to ‘extracellular space’, ‘integral component of plasma membrane’, ‘receptor complex’, ‘extracellular region’, ‘extrinsic component of endosome membrane’, and ‘cell’. The MFs of GO for the jejunal mucosa enriched to ‘progesterone receptor binding’, ‘ATP binding’, ‘ATPase activity’, and ‘L-amino acid transmembrane transporter activity’. Moreover, similar to GO terms, the KEGG pathways for the liver were identified with ‘intestinal immune network for IgA production’, ‘biosynthesis of amino acids’, ‘alanine, aspartate and glutamate metabolism’, ‘cytokine-cytokine receptor interaction’, ‘biosynthesis of antibiotics’, ‘metabolic pathways’, ‘glycine, serine and threonine metabolism’, ‘carbon metabolism’, ‘influenza A’, ‘cell cycle’, ‘DNA replication’, ‘tryptophan metabolism’, ‘propanoate metabolism’, and ‘herpes simplex infection’. The KEGG pathways for the jejunal mucosa were identified with ‘cytokine-cytokine receptor interaction’, ‘protein processing in endoplasmic reticulum’, ‘influenza A’, ‘glutathione metabolism’, ‘NOD-like receptor signaling pathway’, and ‘neuroactive ligand-receptor interaction’. In addition, the results of GSEA for the liver and jejunal mucosa also included KEGG pathways related to energy metabolisms and immune signaling (Additional file 1: Fig. S1).

### Integrative network analysis

**Fig. 5:**
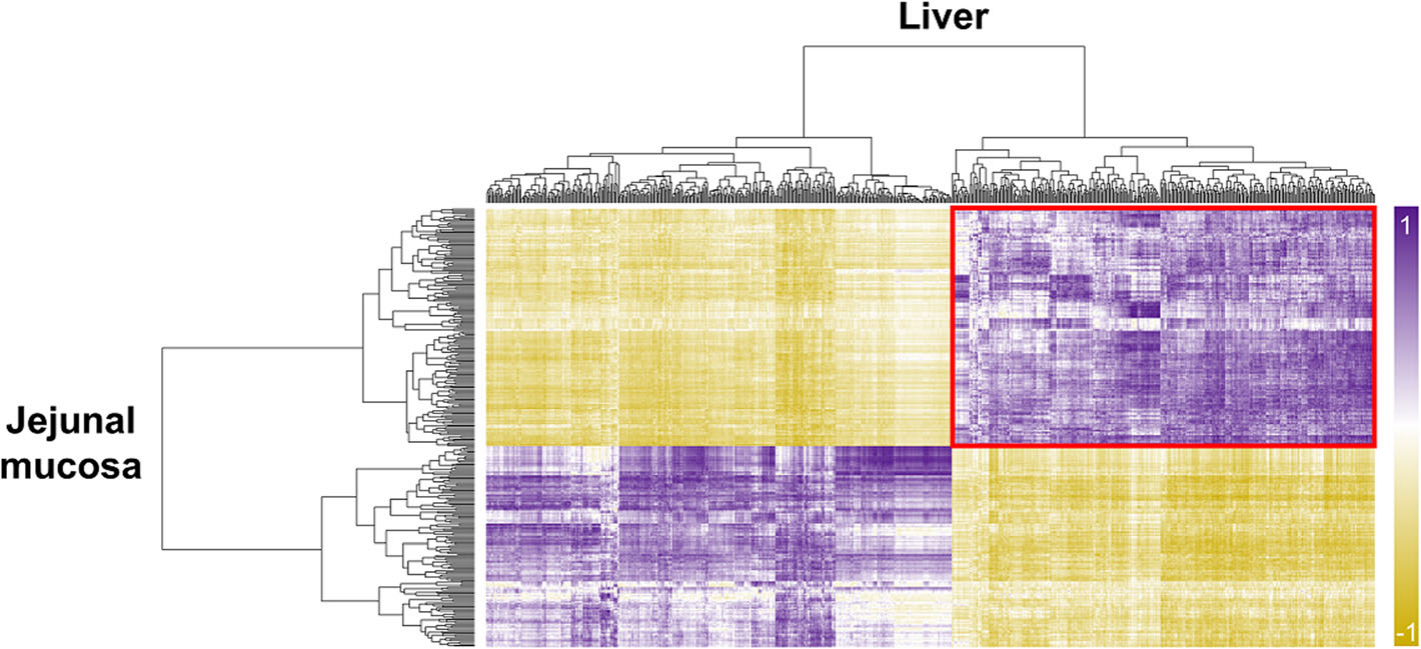
Integrative analyses for the liver and jejunal mucosa. Heatmap for correlation between DEGs in the liver and jejunal mucosa. Red square includes the genes related to energy metabolisms

**Fig. 6:**
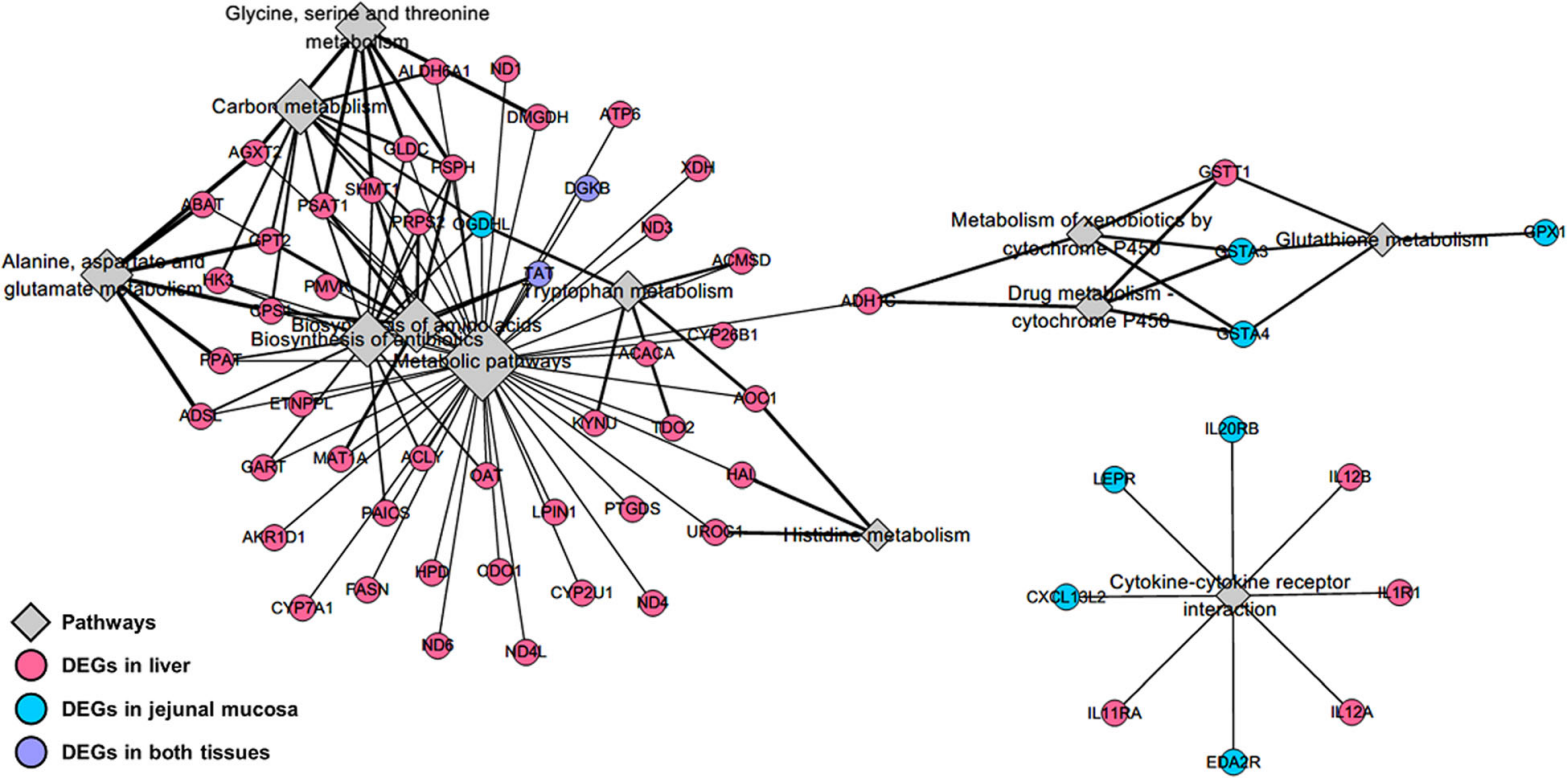
Integrative analyses for the liver and jejunal mucosa. Integrated network for significantly KEGG-enriched pathways with DEGs (*P*-values < 0.1). Diamond size indicates significance and line width indicates fold enrichment in each pathway

To identify functional relationships between the liver and jejunal mucosa tissues, integrated analyses based on DEGs were performed using the construction of correlation matrix and network. The constructed correlation matrix with 737 and 319 DEGs of the liver and jejunal mucosa tissues clearly separated as four clusters. Among four clusters, DEGs in the red squared cluster (liver: 351 and jejunal mucosa: 173 DEGs) included genes related to energy metabolisms and showed a highly positive correlation (Fig. 5). Then, total 494 DEGs (30 overlapping genes) in the red squared cluster were significantly enriched to KEGG pathways related to energy metabolisms (‘metabolic pathways’, ‘biosynthesis of antibiotics’, ‘biosynthesis of amino acids’, ‘carbon metabolism’, ‘alanine, aspartate and glutamate metabolism’, ‘glycine, serine and threonine metabolism’, ‘tryptophan metabolism’, ‘histidine metabolism’), detoxifying metabolisms (‘drug metabolism – cytochrome P450’, ‘metabolism of xenobiotics by cytochrome P450’, and ‘glutathione metabolism’), and immune signaling (‘cytokine-cytokine receptor interaction’). The network for these enriched KEGG pathways was shown in Fig. 6 with DEGs and most of DEGs were involved in the liver.

### Quantitative real-time PCR validation of gene expression

**Fig. 7:**
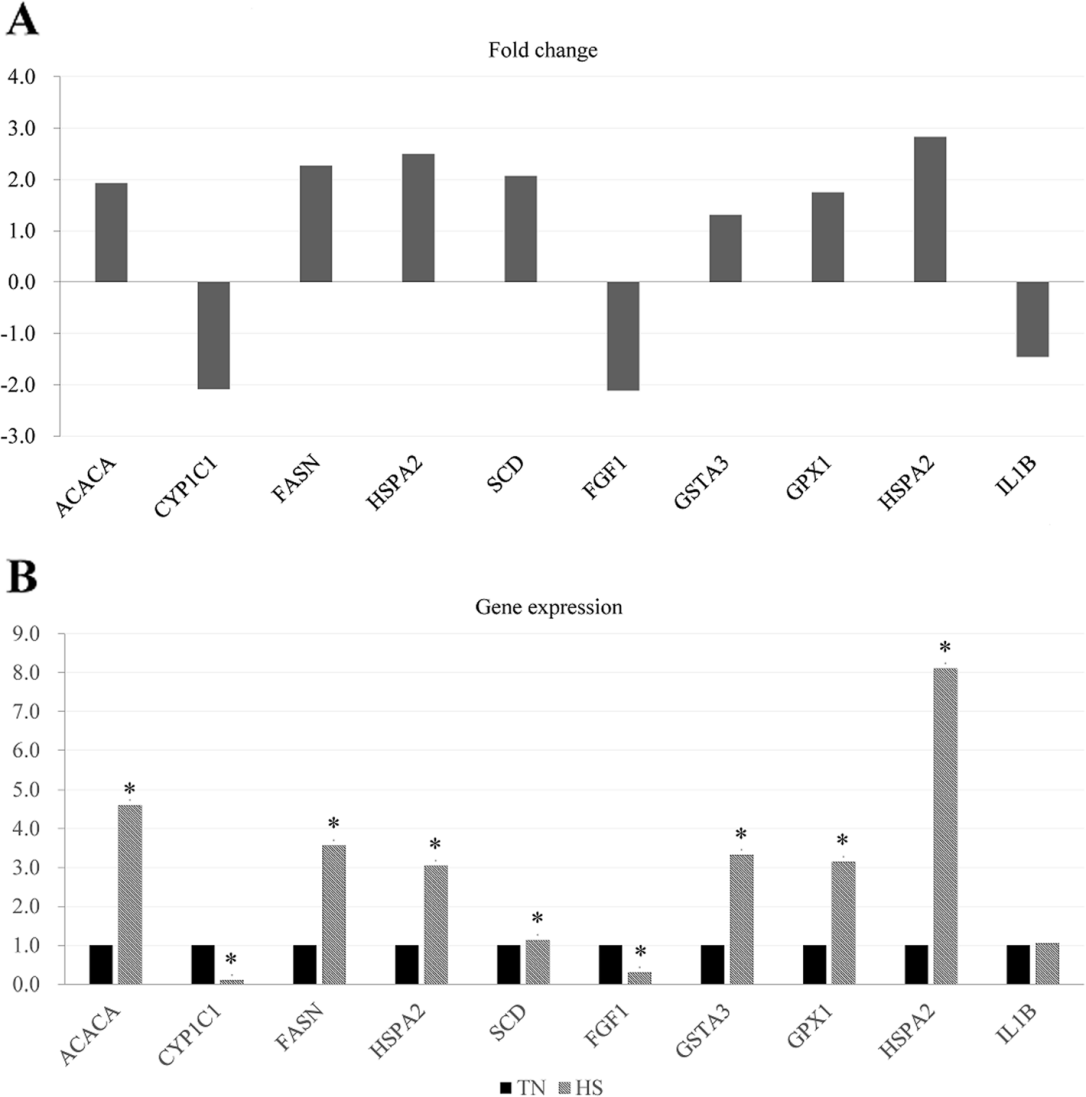
Validation of the differentially expressed genes (DEGs) by RT-PCR (*n* = 8). **A** Comparison (log_2_ fold change) of the RNA-seq data of HS group relative to TN group. **B** Individual variability of validated DEGs in RT-PCR between the TN and HS groups. TN, thermoneutral zone; HS, heat stress. * indicates *P* < 0.05

We selected five DEGs (*HSPA2, CYP1C1, FASN, SCD*, and *ACACA*) in the hepatic tissues and five DEGs (*HSPA2, GPX1, GSTA3, IL1B*, and *FGF1*) in the jejunal mucosa for the validation by qPCR (Fig. 7). All genes showed the similar expression pattern in both of qPCR and RNA-seq. These results demonstrated the reliability of our RNA-seq data and confirmed the accuracy of the identified transcripts.

## Discussion

### Growth performance and feather corticosterone

The HS conditions due to the rapid global warming is considered a significant problem in the current poultry industry. It has been reported that HS conditions lead to a considerable economic loss of poultry production by decreased growth performance and health of poultry [22–24]. Similar result was also observed in the present study. This decrease in the growth performance by HS conditions has been attributed to various physiological and behavioral factors such as a poor appetite [25], less efficiency of nutrients’ adsorption and utilization in the body [26, 27], and impaired health by endocrine disorders, systemic immune dysregulation, and oxidative damage [28–30].

The feather CORT concentrations of poultry have been recently used as a potential biomarker to various stressors [31]. Increased feather CORT concentrations of birds exposed to various stressors (e.g., high stocking density or HS conditions) have been reported previously [32], which agree with our current observation. Therefore, it is appreciated that the HS conditions used in the present study were properly simulated to analyze the integrated transcriptome of the liver and jejunal mucosa of broiler chickens exposed to HS.

## Transcriptome in the liver

The liver is a multipurpose organ involved in bile secretion, nutrient metabolism, and detoxifications [10]. The liver is also involved in immune responses by producing acute-phase protein when animals experience with high inflammatory insults [33]. Therefore, the liver is considered a target metabolic organ as affected primarily by various inherent animal factors and external environmental factors.

Broiler chickens raised under HS conditions are reported to experience a variety of abnormal nutrient metabolism in the body [34–36]. For instance, heat-stressed broiler chickens generally show decreased feed intake (e.g., low nutrient and energy intake), but require more nutrients and energy [23]. It has been reported, however, that heat-stressed broiler chickens do not efficiently utilize body fat as an energy source and conversely exhibit increased fat accumulation in the body [34–37]. In addition, heat-stressed broiler chickens are reported to promote protein catabolism by increasing rate of muscle degradation and amino acid oxidation [38], possibly for supporting increased nutrient and energy requirements of animals [39]. As observed in the previous experiments with heat-stressed broiler chickens, many of these metabolic alterations were confirmed by our hepatic transcriptome results.

Based on the hepatic transcriptomic profiles, broiler chickens raised under HS conditions were shown to alter protein metabolisms, and this functional change may be the reason for decreased growth performance and protein retention as previously observed in heat-stressed broiler chickens.

**Fig. 8:**
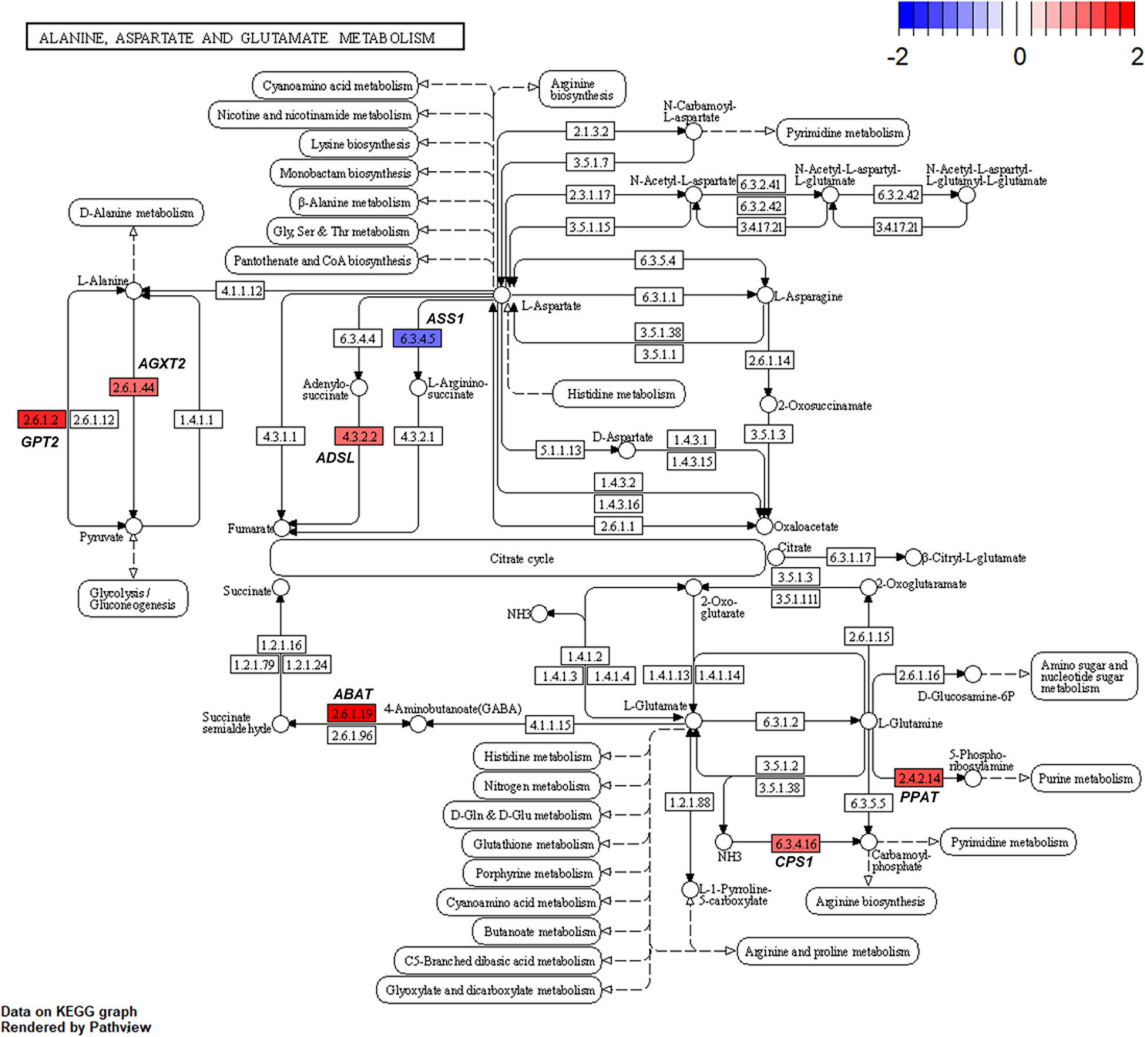
Gene modulations of the liver tissue in ‘alanine-aspartate-glutamate metabolism’ pathway is presented as log_2_ fold change values

**Fig. 9:**
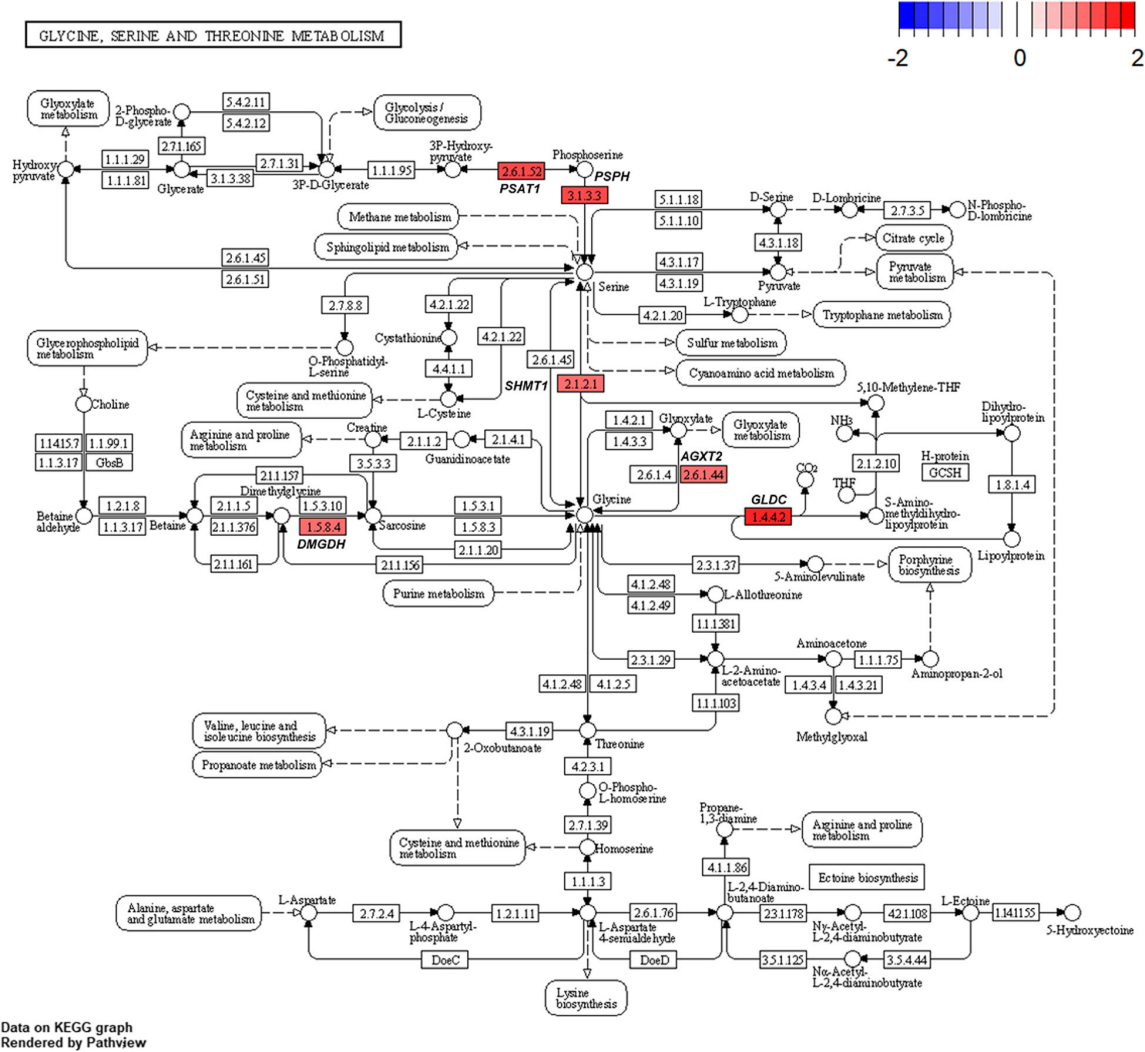
Gene modulations of the liver tissue in ‘glycine-serine-threonine metabolism’ pathway is presented as log_2_ fold change values

**Fig. 10:**
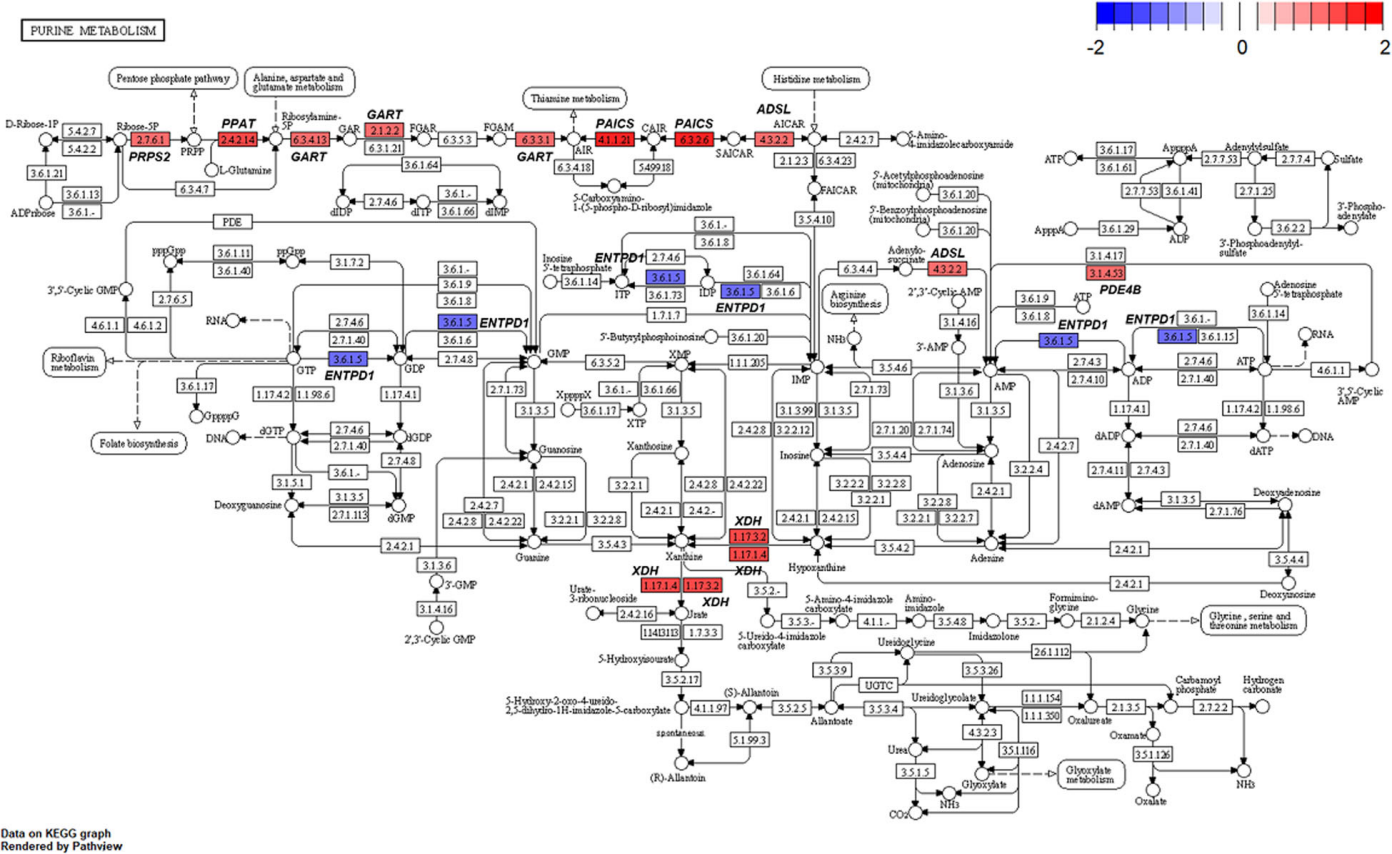
Gene modulations of the liver tissue in ‘purine metabolism’ pathway is presented as log_2_ fold change values

Several genes related to ‘alanine-aspartate-glutamate metabolism’ (Fig. 8) and ‘glycine-serine-threonine metabolism’ (Fig. 9) were altered by HS conditions. Glutamic-pyruvic transaminase 2 (*GPT2*) was up-regulated in the HS conditions. The *GPT2* is one of the primary aminotransferases in poultry and plays a role in maintaining liver health and homeostasis [40]. The *GPT2* catalyzes the reversible transamination between alanine and 2-oxoglutarate to form pyruvate and glutamate [41]. Serine hydroxymethyltransferase 1 (*SHMT1*) was also up-regulated in the HS conditions. This enzyme is known to be associated with cellular one-carbon metabolism by interconversion of glycine and serine [42]. Glycine is easily converted to serine and the converted serine is used for pyruvate production, which may be act as a precursor of gluconeogenesis [43]. In addition, glycine decarboxylase (*GLDC*) was up-regulated in the HS conditions and this enzyme catalyzes direct oxidation of glycine and the production of NH_3_. It appears, therefore, that broiler chickens raised under HS conditions is likely shown to increase glycine oxidation, and therefore, promotes NH_3_ production as a N waste from amino acid oxidation. In poultry, uric acids are the major excretory molecules from the N waste and uric acids are synthesized via purine metabolism in the liver [44]. In the current experiment, genes related to purine metabolism were also up-regulated. Glutamine phosphoribosyl pyrophosphate amidotransferase (*PPAT*), which functions the incorporation of glutamine in the synthesis of purine ring [44], and phosphoribosyl glycinamide synthetase (*GART*), which functions the incorporation of glycine in purine synthesis [44], were highly expressed in the HS conditions. Phosphoribosyl pyrophosphate synthetase 2 (*PRPS2*) is another gene involved in the purine metabolism [45] and this gene was also up-regulated. These altered expressions of several genes related to ‘biosynthesis of amino acids’ and ‘purine metabolism’ (Fig. 10) may indicate that broiler chickens raised under HS conditions experience increased amino acid oxidation in the liver possibly for energy or glucose supply, which accompanies with increased uric acid production via purine metabolism in the liver. It is likely that increased amino acid oxidation in heat-stressed broiler chickens results in decreased protein retention and the subsequent growth performance.

Interestingly, some genes related to urea cycle were also altered in the HS conditions. It has been known that poultry has deficient enzymes involved in the hepatic urea cycle, and therefore, poultry produces the uric acid as a N disposal molecule rather than urea, which is the N disposal molecule in the mammals [46]. In the current study, however, carbamoyl-phosphate synthase 1 (*CPS1*), which has been known to be little expressed in the poultry [47], was up-regulated, whereas argininosuccinate synthase 1 (*ASS1*), which has a very small expression activity in the poultry [48], was down-regulated in the HS conditions. We cannot find any clear reason why these 2 uncommon genes in the poultry were altered by HS conditions; however, it may be also related to detoxification of N as a consequence of enhanced amino acid oxidation. Further researches are required to identify the function of these genes in poultry, particularly under HS conditions.

In poultry, the liver is the main organ for de novo lipogenesis [49]. Several previous studies reported that chronic HS can facilitate fat synthesis and deposition in broiler chickens [34–37]. In the present study, fat metabolism such as fat synthesis and degradation was altered in the HS conditions. Genes related to fat synthesis (*FASN*, acetyl-CoA carboxylase [*ACC*], *SCD*) were up-regulated by more than 2-fold in broiler chickens raised under HS conditions. The *FASN* is a key enzyme catalyzing the synthesis of long-chain fatty acids through the condensation of acetyl-CoA and malonyl-CoA in de novo lipogenesis [50]. The *ACC*, which is the rate-limiting enzyme in the initiation of fatty acid synthesis, catalyzes the carboxylation of acetyl-CoA to malonyl-CoA. Malonyl-CoA synthesized by *ACC* is a critical regulator of enhancing fatty acid synthesis but decreasing β-oxidation of fatty acids [50]. In addition, *SCD* is an essential enzyme to convert palmitic acids and stearic acids, which are the end product of de novo lipogenesis in the body, to palmitoleic acid and oleic acids, respectively [50]. Interestingly, the acyl-CoA thioesterase 7 (*ACOT7*) gene, which is related to ‘biosynthesis of unsaturated fatty acids’ (Additional file 2: Fig. S2.) pathway, was down-regulated in the HS conditions. The *ACOT7* is a group of enzymes that catalyze the hydrolysis of acyl-CoAs to the free fatty acid and coenzyme A (CoASH), providing the potential to regulate intracellular levels of acyl-CoAs, free fatty acids, and CoASH [51], although its clear mechanism is not identified. These results may indicate that heat-stressed broiler chickens undergo with impaired fatty acid breakdown and its subsequent utilization. Therefore, these up-regulation of lipogenesis activity in the liver clearly indicates that broiler chickens raised under HS conditions exhibit abnormal fat synthesis in the liver. Moreover, it is known that the primary source of de novo lipogenesis in poultry is glucose [52]. Therefore, it can be speculated that heat-stressed broiler chickens may utilize a large portion of dietary glucose for fatty acid synthesis, which may lead to a decrease in glucose availability and energy production from the glucose. We suggest that the short of glucose and a concomitant decrease in energy synthesis from glucose may stimulate the gluconeogenesis and energy production from amino acid oxidation because fatty acid oxidation as an alternative energy supply is likely decreased in heat-stressed broiler chickens. This suggestion is supported by our observation for up-regulated genes related to amino acid oxidation.

In ‘tryptophan metabolism’ (Additional file 3: Fig. S3), the genes of aminocarboxymuconate-semialdehyde decarboxylase (*ACMSD*), kynureninase (*KYNU*), and tryptophan 2,3-dioxygenase (*TDO2*) were up-regulated in the HS conditions. These genes are related to niacin synthetic pathway from tryptophan, and therefore, up-regulation of these genes in heat-stressed broiler chickens may indicate increasing activity of converting tryptophan to niacin for supporting increasing requirement of niacin. Niacin is a precursor molecule of nicotinamide adenine dinucleotide (NAD) or nicotinamide adenine dinucleotide phosphate (NADP), which are involved in oxidation and reduction of various nutrients [50]. The reason for increasing niacin production from tryptophan and thus its requirement is likely due to increasing amino acid oxidation and fatty acid synthesis, which require the supply of NAD and NADP as a cofactor.

Increasing synthesis of niacin from tryptophan may also decrease tryptophan utilization for other purposes such as body protein synthesis and immune-related molecules [53]. In addition, tryptophan has an important physiological role in relation to stress response because tryptophan is a precursor of serotonin, which is known to regulate the central nerve systems via suppression of dopamine [54]. The serotonergic projection from the dorsal raphe extends to the substantia nigra and interferes with the firing of the dopamine neurons [54]. Stimulation of the serotonin fiber of the dorsal raphe releases serotonin in the substantia nigra, and this liberated serotonin reduces the firing rate of dopamine neurons, thereby inhibiting dopamine-mediated behavior [55]. It has been reported that feeding diets containing additional tryptophan can increase serotonin synthesis in poultry, and therefore, ameliorates stress responses in poultry [56]. Therefore, our result may provide the reason why additional supplementation of dietary tryptophan show a beneficial effect on poultry exposed to various stressful environments. Vitamin B_6_ is a coenzyme for the conversion of tryptophan to serotonin. However, no genes or pathways associated with vitamin B metabolism were significantly altered by HS conditions. Further researches for the cross-relationship among tryptophan, niacin, and serotonin in heat-stressed animals are required.

In ‘oxidative phosphorylation’ pathway (Additional file 4: Fig. S4), six genes (NADH dehydrogenase subunit 3 [*ND3*], NADH-ubiquinone oxidoreductase chain 4 L [*ND4L*], ATP synthase F0 subunit 6 [*ATP6*], NADH dehydrogenase subunit 1 [*ND1*], NADH dehydrogenase subunit 4 [*ND4*], and NADH dehydrogenase subunit 6 [*ND6*]) were up-regulated in the HS conditions. This finding demonstrates that HS conditions promote the activity of energy production via the electron transport chain (ETC). This increased energy production activity may indicate that heat-stressed broiler chickens show increased energy requirement because it has been reported that increased ETC activity are often followed by increased energy requirements in animals [57]. Moreover, it should be noted that increased ETC activity is highly associated with increasing production of reactive oxygen species [58] because possible inefficiency of electron transfer to O_2_ as a final electron acceptor is the main reason for ROS production in the animal [58]. Previous experiments have reported that heat-stressed animals show increased oxidative stress [59], and therefore, additional supplementation of antioxidants in diets is required to decrease oxidative stress in the animal. Therefore, our findings suggest that increased oxidative stress observed in heat-stressed animals may be due partly to the increasing ETC activity to support increased their energy requirement.

The HS conditions are reported to adversely affect immune responses in broiler chickens [23]. In the liver of heat-stressed broiler chickens, seven genes (C-C motif chemokine receptor 10 [*CCR10*], interleukin 21 receptor [*IL21R*], C-C motif chemokine receptor 7 [*CCR7*], interleukin 18 receptor 1 [*IL18R1*], C-C motif chemokine ligand 5 [*CCL5*], X-C motif chemokine receptor 1 [*XCR1*], and interleukin 10 receptor subunit alpha [*IL10RA*]) was down-regulated in ‘cytokine-cytokine receptor interaction’ (Additional file 5: Fig. S5). The inducible T cell costimulatory (*ICOS*) was also down-regulated in the liver. In an inflammatory reaction, various chemokines play a role in blood movements to inflammatory sites, and therefore, increases accessibility of immune cells to inflammation [60]. Chemokine is classified into four types; CXC, CC, C, and CX3C chemokine (CXC chemokine, separated by one amino acid between the first two cysteine groups; CC chemokine, two cysteine are connected; C chemokine, only one cysteine; CX3C, separated by three different amino acids between the two cysteines) [60]. In this study, CXC and CC chemokines were down-regulated, which indicate that heat-stressed broiler chickens may show decreased anti-inflammatory response. In addition, interleukin (IL) is secreted from leukocytes and plays a role as a key cytokine to regulate the immune response [61]. We found that some interleukin receptors including *IL10*, *IL18*, and *IL21* receptors were down-regulated, which may also indicate that HS conditions decreases immune responses in broiler chickens.

### Transcriptome in the jejunal mucosa

The small intestine is the major organ responsible for the digestion and absorption of dietary nutrients [11]. Enterocytes, the epithelial absorptive cells of the small intestine, represent more than 80% of the mucosal epithelial cell population [62, 63] and show the high intracellular protein turnover and cell proliferation [64]. Furthermore, the mucosa of the small intestine is constantly exposed to various external and harmful environment such as toxins and pathogens, and therefore, the efficient barrier function is necessary to protect the body from the invasion of the toxins and pathogens [12]. The defense mechanisms of the intestinal mucosa include mucous as a chemical defense and a tight junction as a mechanical defense [36]. In addition, the small intestinal tissues play an important role in immune responses in animals because the intestine is continuously exposed to antigens and immuno-stimulating agents derived from the diet and the microorganisms, and thus, it is considered the port of the entry for many antigens [65]. As a result, the small intestine is considered a metabolically active organ in the body.

Previous experiments have reported that heat-stressed animals showed decreased nutrient digestibility and absorption possibly owing to impaired mucosal structure and integrity [27] and decreased nutrient absorption and transport [66]. It has been reported that heat-stressed animals showed down-regulated genes associated with nutrient absorption and transport [67]. Thus, it was expected that broiler chickens raised under HS conditions in the current study showed decreased expressions of genes related to nutrient digestion, absorption, and transport in the jejunal mucosa. However, no significant alterations in the related genes were identified in the jejunal mucosa. The reason is not clear; however, it may be related to differences in animals, diets, and environment among experiments. Limitation in the sensitivity of RNA-seq for verifying differences in expression of target genes may be also the reason [68].

The HS conditions can induce pathological damages to intestinal defense systems in the poultry. Diversion of the blood flow from the intestine to the skin is observed in heat-stressed animals, and therefore, intestinal blood supply is decreased by HS [12]. This reduced blood supply causes the mucosal hypoxia, which results in increased oxidative damages and cell dysfunctions, and subsequently decreases intestinal defense systems [12]. However, we found no significant alterations in the expression of genes related to tight junction barrier function and mucous production that play a key role in intestinal defense systems. The reason for this result may also be due to less sensitivity of RNA-Seq to detect the specific gene expression [68]. However, gene expression of several heat shock protein (HSP) including HSP40 (*DNAJA1*), HSP70 (*HSPA2* and *HSPA8*), HSP90 (*HSP90AA1*), and HSP110 were up-regulated in the jejunal mucosa of heat-stressed chickens (Additional file 6: Fig. S6). The HSP is known to be important for the survival of stressed cells and stabilization of cellular hemostasis by preventing protein misfolding and removing damaged proteins [69, 70]. Therefore, it is suggested that HS conditions may increase cellular protein damages and decrease the ability to maintain cellular homeostasis in the jejunal mucosa.

Furthermore, heat-stressed chickens showed up-regulation of genes related to glutathione metabolism. Glutathione peroxidase (*GPX1*) is an important antioxidant enzyme to convert toxic hydrogen peroxide to water by oxidation of glutathione in the cell [71]. Increased expression of *GPX1* in the jejunal mucosa may indicate increased oxidative stress in the intestinal cells by HS conditions. In addition, the expression of glutathione-S-transferases (*GSTA3* and *GSTA4*) was up-regulated in HS conditions (Additional file 7: Fig. S7). The glutathione-S-transferases (*GST*) is known to play a role in detoxifying various cellular toxins such as xenobiotics, and therefore, improves the cellular integrity and function [72]. It has been reported that increased expression of *GST* may be an indicator of cellular damage and dysfunction [73], which suggest that HS conditions may impair mucosal integrity and functions. Previous experiments also reported that heat-stressed animals showed increased expression of *GPX1* and *GST*, which was in accordance with our findings.

It has been reported that immune responses in the small intestine are affected by HS conditions [74]. Increased inflammatory responses in the intestinal tissues may impair intestinal immune responses of heat-stressed animals [74]. As observed in the liver, interleukin-1 beta (*IL1B*) and interleukin-1 receptor type 2 (*IL1R2*) were down-regulated in the jejunal mucosa of heat-stressed broiler chickens (Additional file 8: Fig. S8). Interleukins are a group of cytokines (secreted proteins and signaling molecules) that are first expressed on white blood cells (white blood cells). Especially, *IL1B* is a potent mediator in response to infection and injury [75]. Therefore, it is suggested that immune systems in the jejunal mucosa is decreased in heat-stressed poultry, which was also observed in the liver of heat-stressed broiler chickens in the current study.

### The core network for the liver and jejunal mucosa

Identification of DEGs in interactive networks and analysis of their related cellular pathway is a key step toward a better understanding of the functional crosstalk among various tissues [18]. In the current study, we investigated an integrated gene co-expression network between the liver and jejunal mucosa of broiler chickens raised under HS conditions and identified several key metabolic pathways bridging physiological and functional relationships between these directly connected tissues.

As a result of integrated transcriptome analysis, genes related to energy metabolism are highly and positively correlated between the liver and jejunal mucosa (Fig. 5). A considerable connectivity was observed between the liver and jejunal mucosal functions with various pathway terminologies including glutathione metabolism, xenobiotic metabolism, carbon metabolism, and several amino acid metabolisms (Fig. 6). The intestinal tissues are known to require the largest amounts of energy and amino acids in the animal body because of high turn-over rate of mucosal cells and its continuous reconstruction [76]. In addition, it is well-known that HS impairs intestinal health due to high inflammatory reactions caused by increasing permeation of toxins and pathogens from the intestinal lumen [77]. We also observed increased expression of genes that are enriched in the detoxifying metabolism by cytochrome P450 and glutathione metabolism in the jejunal tissues, which may indirectly indicate impaired intestinal health such as decreased integrity and increased oxidative stress. It appears that decreased intestinal structure and integrity as well as increased oxidative stress result in an increasing demand for the fast cure and recovery of intestinal tissues, which requires a large amount of energy and nutrients such as amino acids. In the current experiment, several amino acid metabolisms and biosynthesis of amino acids and antibiotics were identified based on the integrated transcriptome in the liver. Therefore, we speculate that increased amino acid oxidation and biosynthesis of amino acids in the liver are likely related to increased needs of energy and amino acids in the jejunal tissues of heat-stressed broiler chickens. Summarizing, the core network connecting the transcriptome of the liver and jejunal mucosa may reveal that increased requirement of energy and amino acids in the jejunal mucosa as a consequence of HS conditions is supported by increased oxidation and synthesis of amino acids in the liver.

## Conclusions

Broiler chickens raised under HS conditions show decreased growth performance and increased CORT concentrations in the feather. Based on the transcriptomic analysis in the liver, HS conditions increases amino acid oxidation to support increased energy production, whereas glucose is likely prioritized for fatty acid synthesis. Interestingly, tryptophan oxidation for niacin synthesis is increased, which may limit serotonin synthesis, and therefore, aggravates stress responses. In the jejunal mucosa, increased detoxification activity and antioxidant system are identified, indicating impaired intestinal health and increased oxidative stress. Integrated transcriptome analysis between the liver and jejunal mucosa demonstrates the core genes in the network that may modulate the energy metabolism. The core network genes enriched in the metabolic pathways indicate that increased requirement of energy and amino acids in the jejunal mucosa of broiler chickens by HS conditions is likely supported by increased oxidation and synthesis of amino acids in the liver.

## Supplementary Information

**Additional file 1: Table S1.** RNA quality score of broiler chickens raised under thermoneutral (TN) or heat stress (HS) conditions.1**. Table S2.** Primers used for quantitative RT-PCR**. Table S3.** Overview of data processing of broiler chickens raised under thermoneutral zone (TN) or heat stress (HS) conditions. **Fig. S1.** Bubble plots for gene set-enrichment analyses (GSEA) of each tissue. (**A**) GSEA for liver tissue. (**B**) GSEA for jejunal mucosa tissue. Cut-off is *P*-values < 0.05 and size indicates the number of genes corresponding to each pathway.

**Additional file 2: Fig. S2.** Gene modulations of the liver tissue in ‘biosynthesis of unsaturated fatty acids’ pathway is presented as log_2_ fold change values.

**Additional file 3: Fig. S3.** Gene modulations of the liver tissue in ‘tryptophan metabolism*’* pathway is presented as log_2_ fold change values.

**Additional file 4: Fig. S4.** Gene modulations of the liver tissue in ‘oxidative phosphorylation’ pathway is presented as log_2_ fold change values.

**Additional file 5: Fig. S5.** Gene modulations of the liver tissue in ‘cytokine-cytokine receptor interaction’ pathway is presented as log_2_ fold change values.

**Additional file 6: Fig. S6.** Gene modulations of the jejunal mucosa tissue in ‘protein processing in endoplasmic reticulum’ pathway is presented as log_2_ fold change values.

**Additional file 7: Fig. S7.** Gene modulations of the jejunal mucosa tissue in ‘glutathione metabolism’ pathway is presented as log_2_ fold change values.

**Additional file 8: Fig. S8.** Gene modulations of the jejunal mucosa tissue in ‘cytokine-cytokine receptor interaction’ pathway is presented as log_2_ fold change values.

## Data Availability

The datasets generated and/or analyzed during the present study are only available from the corresponding author on reasonable request.

## Abbreviations

ACC: Acetyl-CoA carboxylase
ACMSD: Aminocarboxymuconate-semialdehyde decarboxylase
ACOT7: Acyl-CoA thioesterase 7
ASS1: Argininosuccinate synthase 1
BP: Biological process
BW: Body weight
BWG: Body weight gain
CC: Cellular component
CoASH: Coenzyme A
CORT: Corticosterone
CPS1: Carbamoyl-phosphate synthase 1
DAVID: Database for annotation, visualization, and integrated discovery
DEG: Differentially expressed gene
ETC: Electron transport chain
FAS: Fatty acid synthase
FC: Fold-change
FDR: False discovery rate
FE: Feed efficiency
FI: Feed intake
GART: Phosphoribosyl glycinamide synthetase
GLDC: Glycine decarboxylase
GO: Gene ontology
GPT2: Glutamic-pyruvic transaminase 2
GPX1: Glutathione peroxidase
GSEA: Gene set-enrichment analyses
GST: Glutathione-S-transferases
HS: Heat stress
HSP: Heat shock protein
IL: Interleukin
IL1B: Interleukin-1 beta
IL1R2: Interleukin-1 receptor type 2
KEGG: Kyoto encyclopedia of genes and genomes
KYNU: Kynureninase
MDS: Multidimensional scaling
MF: Molecular function
NES: Normalized enrichment score
PPAT: Glutamine phosphoribosyl pyrophosphate amidotransferase
PRPS2: Phosphoribosyl pyrophosphate synthetase 2
RH: Relative humidity
RNA-Seq: RNA sequencing
ROS: Reactive oxygen species
SCD: Stearoyl-CoA desaturase
SHMT1: Serine hydroxymethyltransferase 1
TDO2: Tryptophan 2,3-dioxygenase
TMM: Trimmed mean of M-value
TN: Thermoneutral zone
