# Supplementary figures and images for: Integrated transcriptome analysis for the hepatic and jejunal mucosa tissues of broiler chickens raised under heat stress conditions
Source: J Anim Sci Biotechnol. 2022 Jul 18;13:79. doi: 10.1186/s40104-022-00734-y (PMC9290309; doi:10.1186/s40104-022-00734-y)

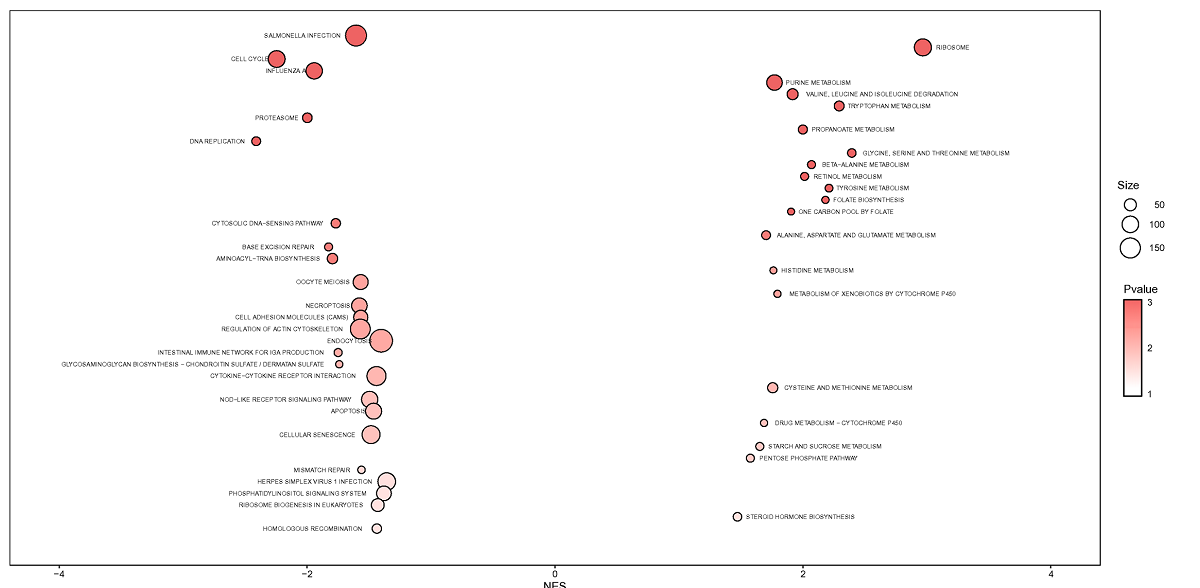

Supplement: Supplementary file 1 — Additional file 1: Table S1. RNA quality score of broiler chickens raised under thermoneutral (TN) or heat stress (HS) conditions.1. Table S2. Primers used for quantitative RT-PCR. Table S3. Overview of data processing of broiler chickens raised under thermoneutral zone (TN) or heat stress (HS) conditions. Fig. S1. Bubble plots for gene set-enrichment analyses (GSEA) of each tissue. (A) GSEA for liver tissue. (B) GSEA for jejunal mucosa tissue. Cut-off is P-values < 0.05 and size indicates the number of genes corresponding to each pathway. [file 40104_2022_734_MOESM1_ESM.zip › Fig. S1A.tif]

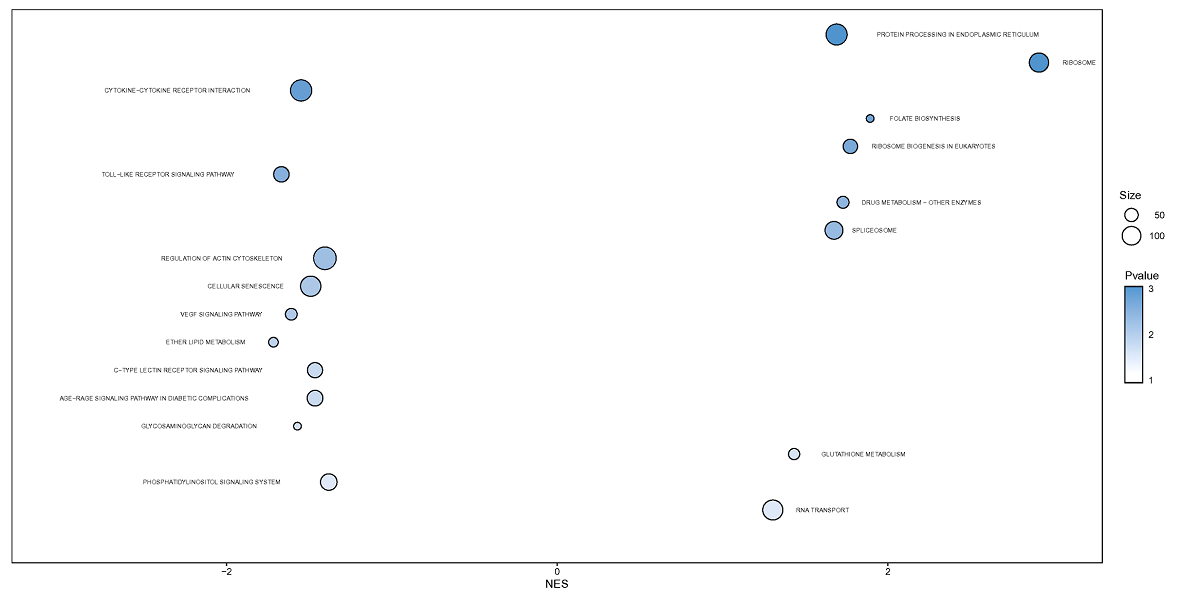

Supplement: Supplementary file 1 — Additional file 1: Table S1. RNA quality score of broiler chickens raised under thermoneutral (TN) or heat stress (HS) conditions.1. Table S2. Primers used for quantitative RT-PCR. Table S3. Overview of data processing of broiler chickens raised under thermoneutral zone (TN) or heat stress (HS) conditions. Fig. S1. Bubble plots for gene set-enrichment analyses (GSEA) of each tissue. (A) GSEA for liver tissue. (B) GSEA for jejunal mucosa tissue. Cut-off is P-values < 0.05 and size indicates the number of genes corresponding to each pathway. [file 40104_2022_734_MOESM1_ESM.zip › Fig. S1B.tif]

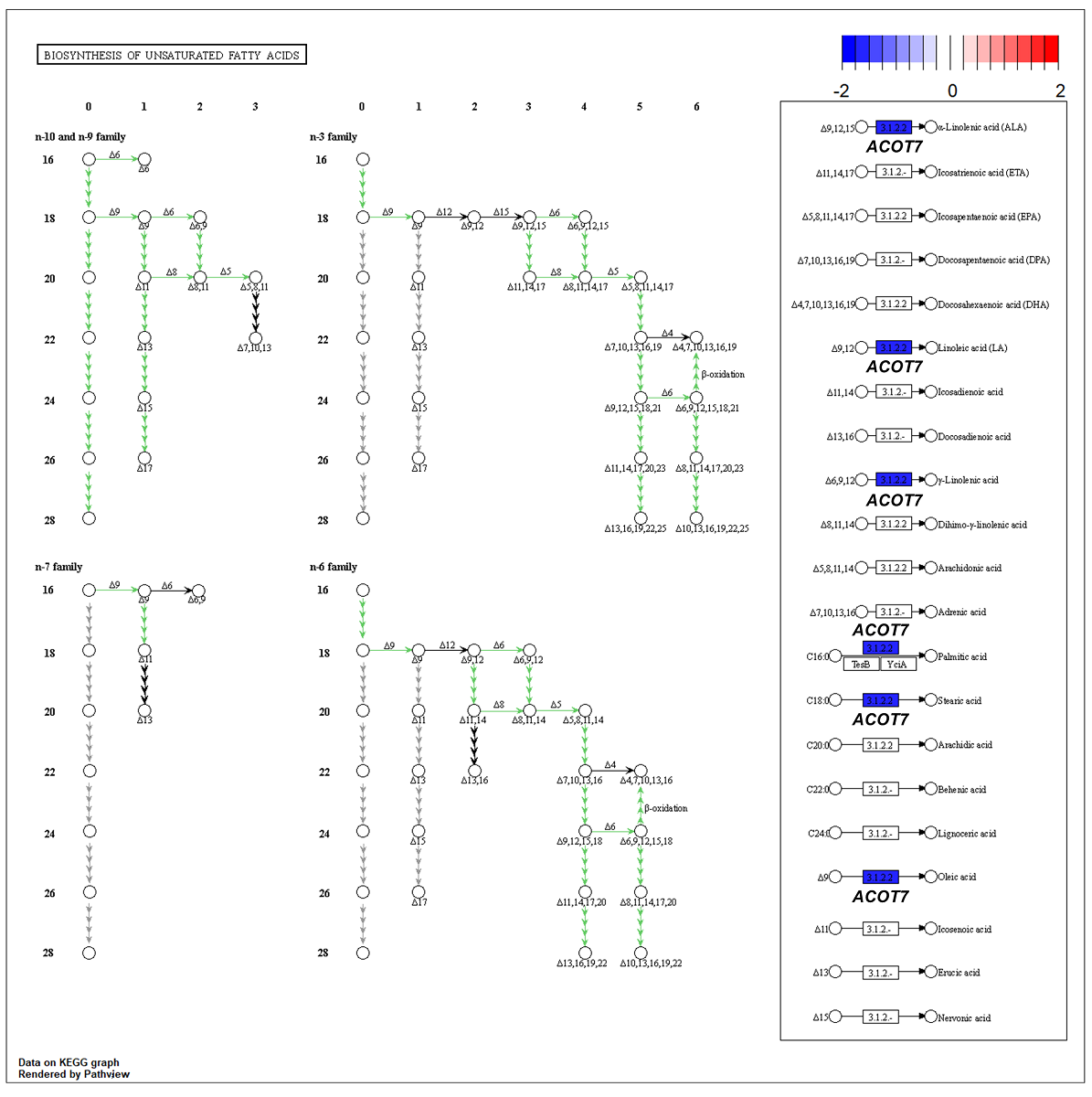

Supplement: Supplementary file 2 — Additional file 2: Fig. S2. Gene modulations of the liver tissue in ‘biosynthesis of unsaturated fatty acids’ pathway is presented as log2 fold change values. [file 40104_2022_734_MOESM2_ESM.tif]

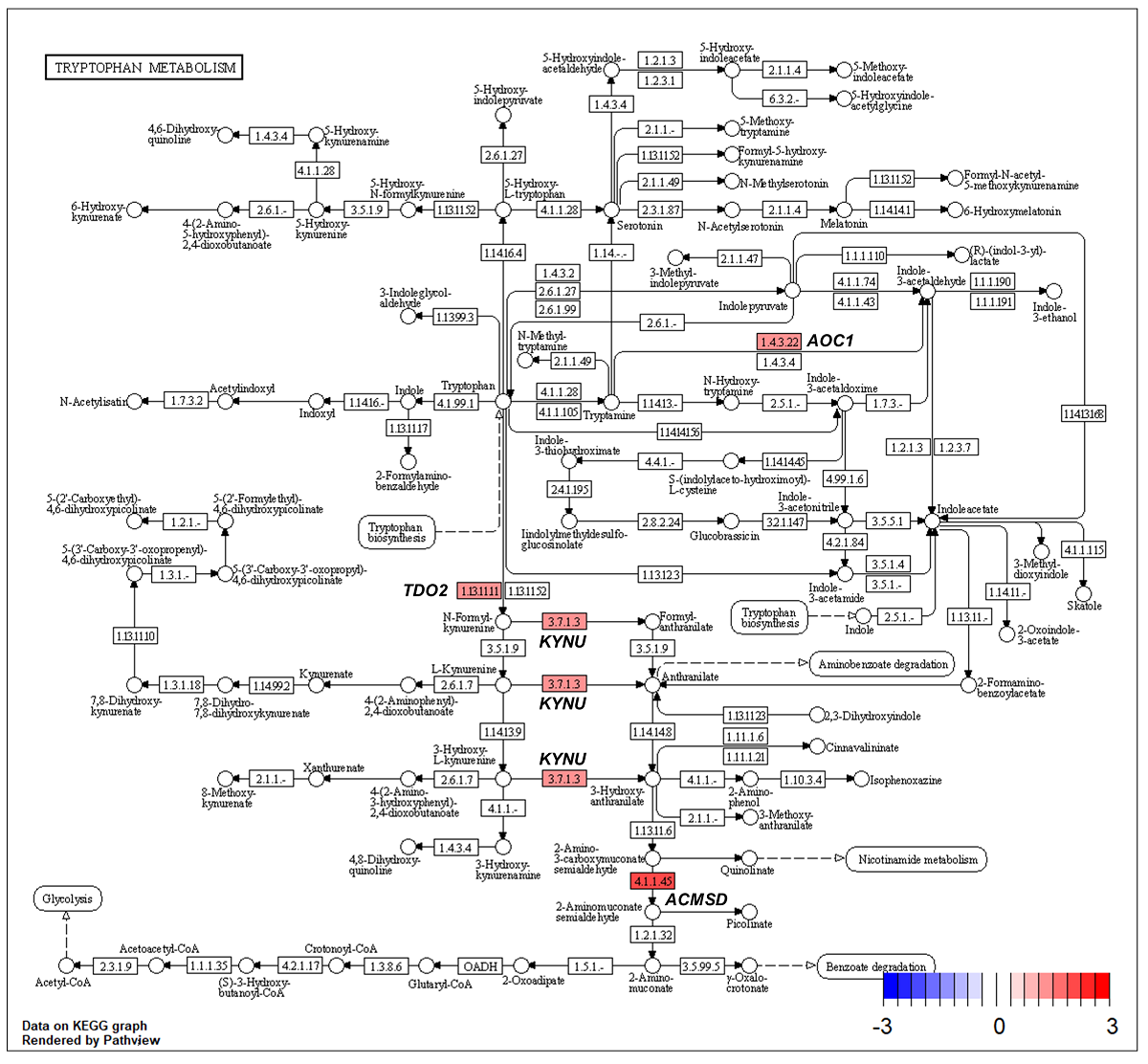

Supplement: Supplementary file 3 — Additional file 3: Fig. S3. Gene modulations of the liver tissue in ‘tryptophan metabolism’ pathway is presented as log2 fold change values. [file 40104_2022_734_MOESM3_ESM.tif]

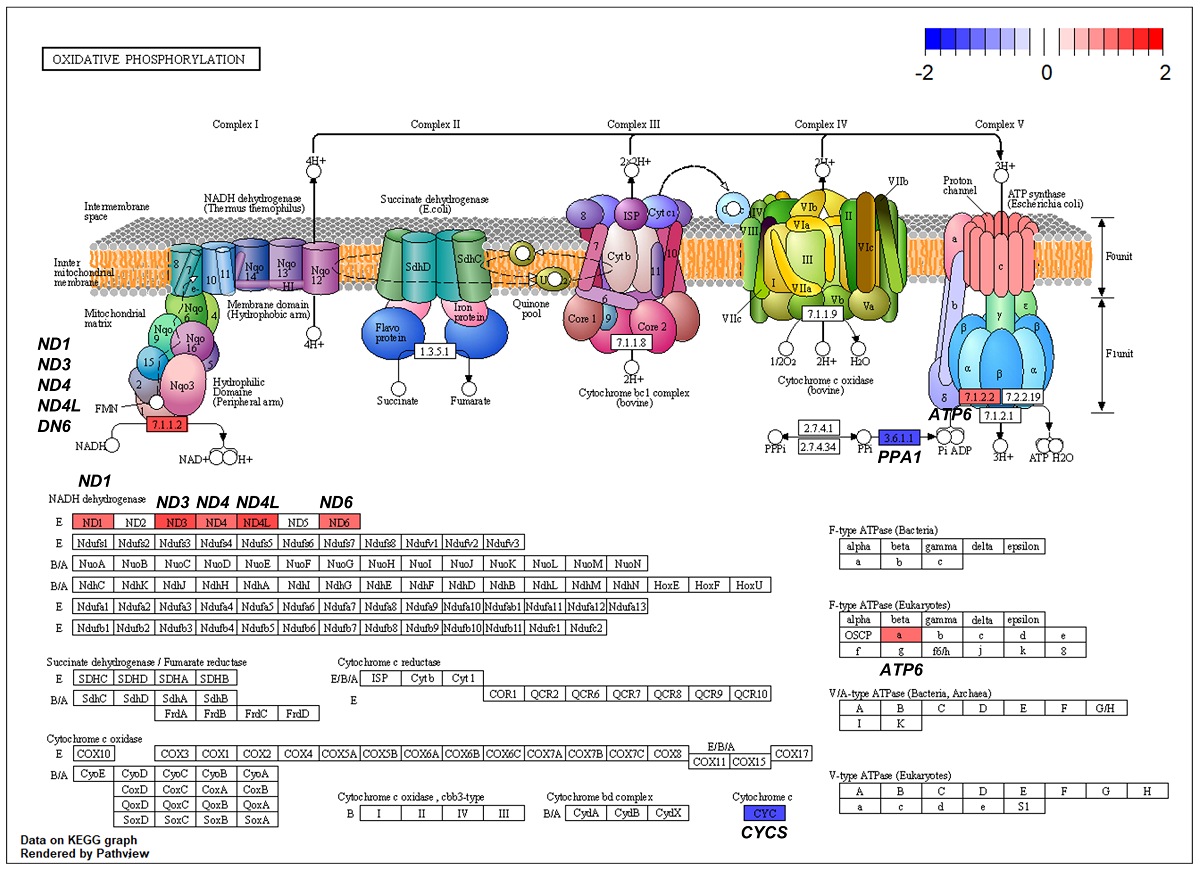

Supplement: Supplementary file 4 — Additional file 4: Fig. S4. Gene modulations of the liver tissue in ‘oxidative phosphorylation’ pathway is presented as log2 fold change values. [file 40104_2022_734_MOESM4_ESM.tif]

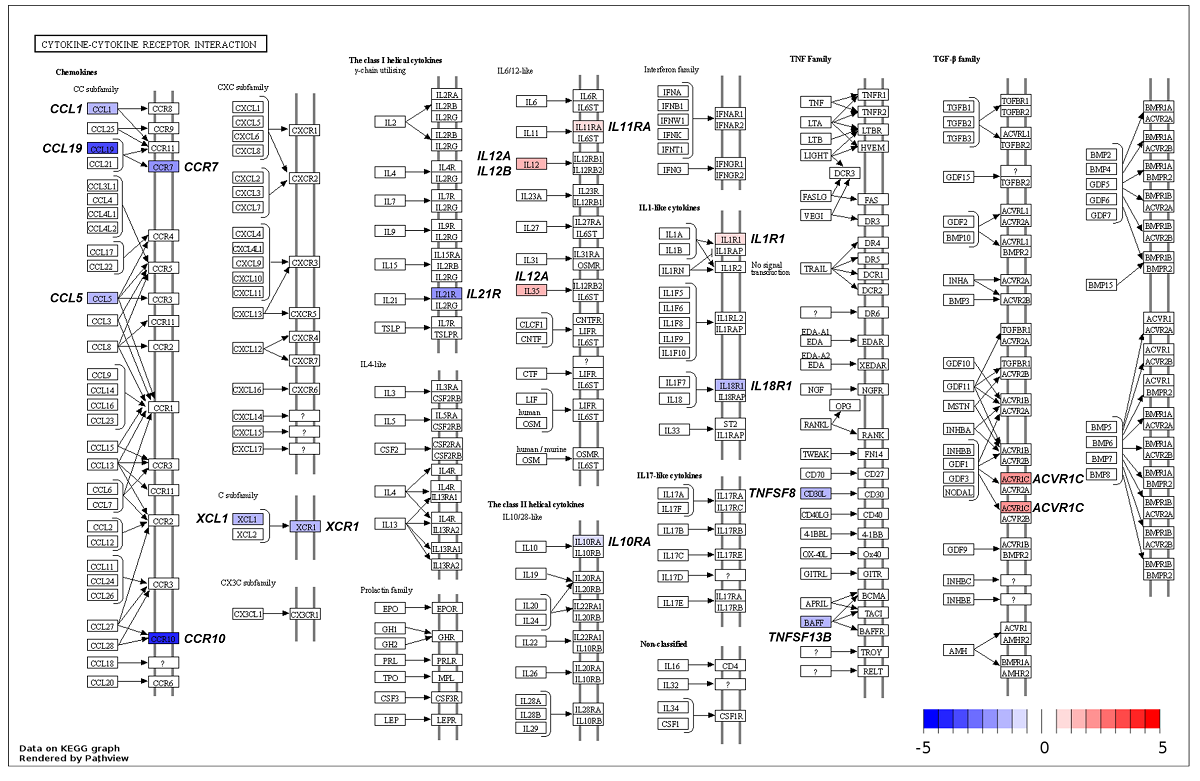

Supplement: Supplementary file 5 — Additional file 5: Fig. S5. Gene modulations of the liver tissue in ‘cytokine-cytokine receptor interaction’ pathway is presented as log2 fold change values. [file 40104_2022_734_MOESM5_ESM.tif]

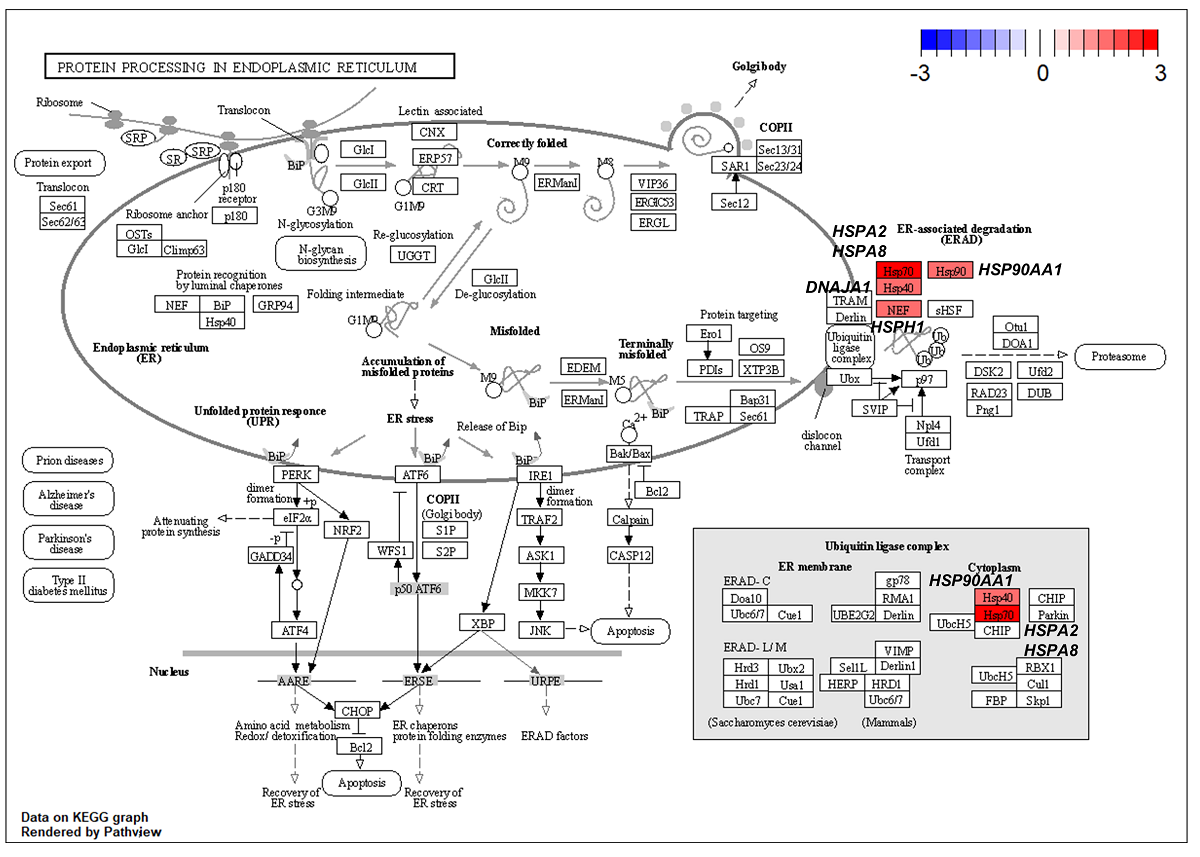

Supplement: Supplementary file 6 — Additional file 6: Fig. S6. Gene modulations of the jejunal mucosa tissue in ‘protein processing in endoplasmic reticulum’ pathway is presented as log2 fold change values. [file 40104_2022_734_MOESM6_ESM.tif]

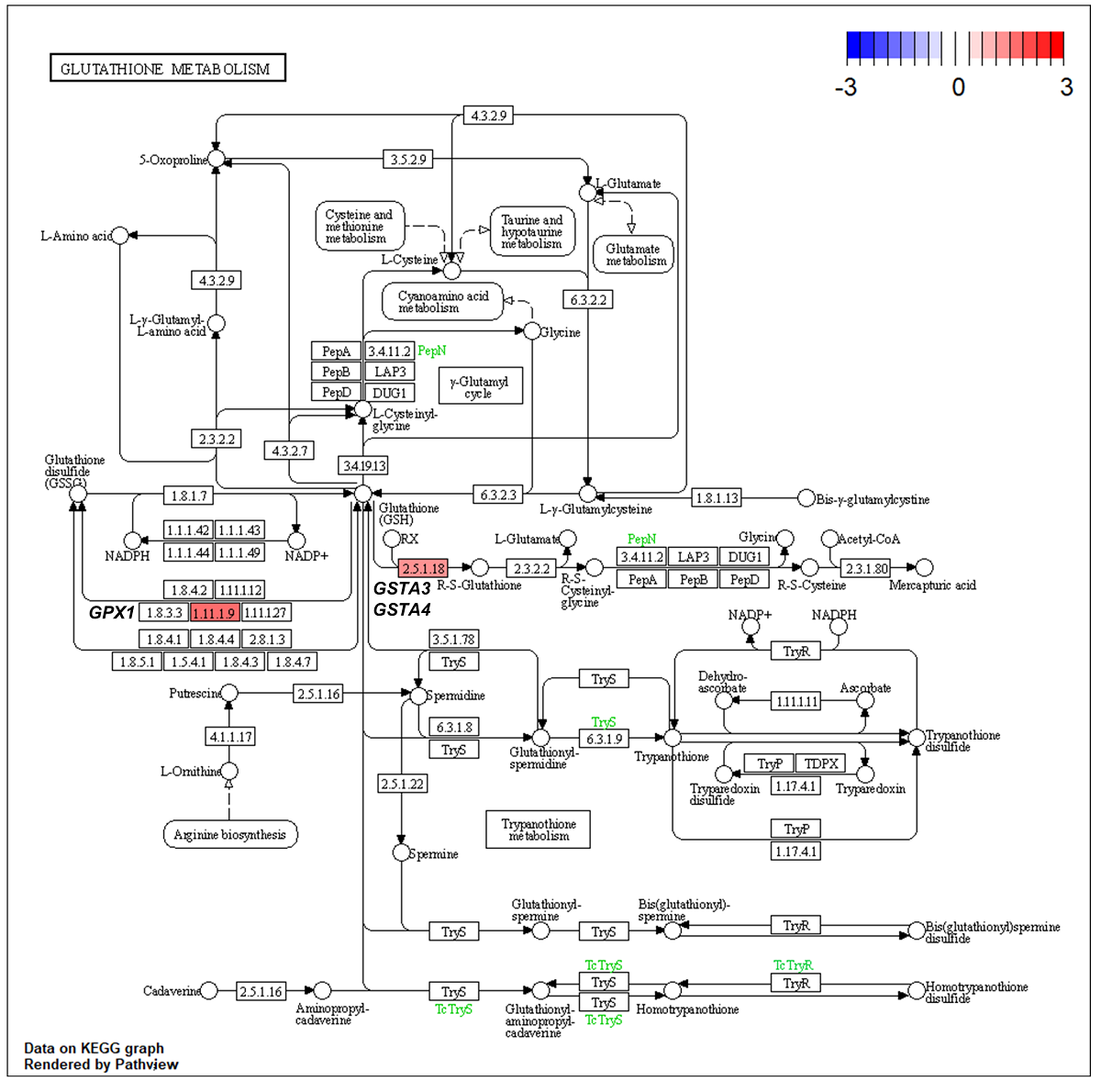

Supplement: Supplementary file 7 — Additional file 7: Fig. S7. Gene modulations of the jejunal mucosa tissue in ‘glutathione metabolism’ pathway is presented as log2 fold change values. [file 40104_2022_734_MOESM7_ESM.tif]

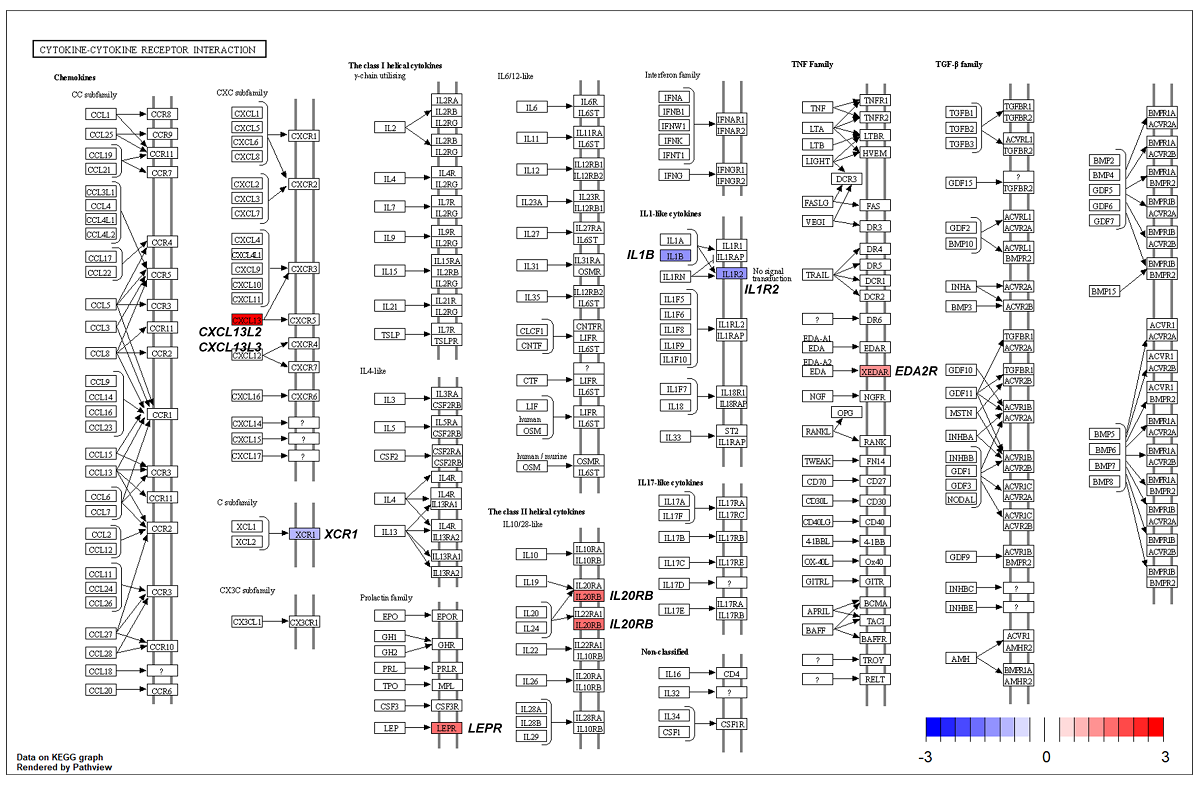

Supplement: Supplementary file 8 — Additional file 8: Fig. S8. Gene modulations of the jejunal mucosa tissue in ‘cytokine-cytokine receptor interaction’ pathway is presented as log2 fold change values. [file 40104_2022_734_MOESM8_ESM.tif]
